# Psychosocial, Clinical, and Knowledge-Based Factors Associated with Self-Care in Heart Failure: A Cross-Sectional Study

**DOI:** 10.3390/healthcare14172772

**Published:** 2026-09-01

**Authors:** Mohammad H. Abuadas, Walid Al-Qerem, Salam Bani Hani, Ahmad M. Al-Bashaireh, Yousef Mohammed Alshahrani, Ghaleb Alharbi, Eidan Alrasheid, Zainab Albikawi, Osama Alkouri, Abdulkareem Alshehri, Fatma Refaat Ahmed, Ahmad Saifan, Mohannad Alkhateeb, Nizar Alsubahi

**Affiliations:** 1 Faculty of Nursing, Yarmouk University, Irbid 21163, Jordan; 2 Faculty of Pharmacy, Al-Zaytoonah University of Jordan, Amman 11733, Jordan; 3 School of Nursing, Irbid National University, Irbid 21163, Jordan; 4 Faculty of Nursing, Philadelphia University, Amman 11118, Jordan; 5 Department of Clinical Technology, Umm Al-Qura University, Makkah 24231, Saudi Arabia; 6 Department of Clinical Pharmacy, College of Pharmacy, Shaqra University, Shaqra 11961, Saudi Arabia; 7 Medical Surgical Nursing Department, College of Nursing, Jouf University, Sakaka 72311, Saudi Arabia; 8 Nursing Department, Ministry of Health, Kuwait City 13001, Kuwait; 9 Advanced Diagnostic and Therapeutic Institute, King Abdulaziz City for Science and Technology (KACST), Jeddah 21589, Saudi Arabia; 10 College of Health Sciences, University of Sharjah, Sharjah 61195, United Arab Emirates; 11 Department of Health Service and Hospital Administration, Faculty of Economics and Administration, King Abdulaziz University, Jeddah 21589, Saudi Arabia; 12 School of Public Health and Community Medicine, Institute of Medicine, University of Gothenburg, 40530 Gothenburg, Sweden; 13 Department of Health Services Research, Care and Public Health Research Institute (CAPHRI), Faculty of Health, Medicine and Life Sciences, Maastricht University, 6200 Maastricht, The Netherlands

**Keywords:** anxiety and depression, heart failure, self-care behaviour, self-management, patient knowledge

## Abstract

Aim: This study examined cross-sectional associations between sociodemographic, clinical, behavioral, knowledge-based, and psychological factors and three behavioral scales of heart failure (HF) self-care: Maintenance, Symptom Perception, and Management. Self-Care Confidence was examined as a separate 10-item outcome. Methods: This cross-sectional study recruited 516 consecutive adults with confirmed HF at two tertiary-care HF clinics in northern Jordan between May and November 2025. Self-care was assessed using the SCHFI v7.2, HF knowledge using the AHFKT, and anxiety and depression using the HADS. Separate multivariable ordinary least-squares regression models estimated adjusted associations with Maintenance, Symptom Perception, Management, and Confidence. Association-rule mining was used to identify descriptive co-occurrence profiles. Results: Median SCHFI scores were 50.00 for Maintenance, 43.48 for Symptom Perception, and 51.52 for Management. All four regression models included 516 participants and showed limited explanatory performance (adjusted R^2^ = 0.076–0.127). Each 10-percentage-point increase in the AHFKT percentage-correct score was associated with higher Maintenance (unstandardized coefficient B = 2.02, 95% CI 0.50–3.54), Symptom Perception (B = 2.26, 95% CI 0.78–3.73), and Management (B = 3.81, 95% CI 2.32–5.30). HADS depression was inversely associated with Maintenance (B = −0.94, 95% CI −1.57 to −0.31), Symptom Perception (B = −1.47, 95% CI −2.10 to −0.83), and Management (B = −1.09, 95% CI −1.70 to −0.48). HADS anxiety was positively associated with Symptom Perception (B = 1.37, 95% CI 0.74–2.00) and Management (B = 0.92, 95% CI 0.33–1.51). Elementary and secondary education were associated with lower Maintenance scores. Current smoking (B = −6.69, 95% CI −10.78 to −2.60) and no prior HF admission (B = −4.32, 95% CI −8.21 to −0.42) were separately associated with lower Symptom Perception. Association-rule mining retained 10 exploratory co-occurrence profiles; the highest-lift profile involved male sex, sleeping difficulty, and an HF knowledge score from 50% to <60% for Management scores < 70 (50/51 participants; confidence = 98.0%; lift = 1.25). For the separate Confidence outcome, no profiles met the same final criteria. Conclusions: Most participants had scores below 70 across the three behavioral scales. Higher HF knowledge was associated with higher scores across self-care maintenance, symptom perception, and self-care management, and higher HADS depression was associated with lower self-care confidence, whereas HADS anxiety was positively associated with self-care confidence; education, smoking, and prior HF admission showed further scale-specific cross-sectional associations.

## 1. Introduction

The condition known as heart failure (HF) constitutes one of the major global public health issues with high morbidity, mortality, and rehospitalization rates despite considerable medical advancements [1]. One of the contributing factors to poor outcomes includes poor self-care in patients [2]. Heart failure self-care comprises complicated behaviors, including adherence to medications, food intake, and fluid restriction, monitoring of symptoms, physical activities, and prompt reactions to changes in symptoms that affect quality of life and prognosis [3]. Modern self-care models, such as the Self-Care of Heart Failure Index (SCHFI), define self-care based on self-care maintenance, symptom perception, self-care management, and self-care confidence [4].

While self-care is highly advised in guidelines for HF, its compliance has remained inadequate across the globe, with rates of non-compliance touching 60% in recent studies [4,5]. More recent studies are starting to reveal the complexity of factors associated with the self-care of HF, including knowledge, sociodemographic factors, psychological issues, and social support networks [6,7]. Factors such as lack of knowledge, health literacy, financial issues, and psychological well-being might affect self-care. Moreover, it is suggested that knowledge alone does not suffice; people must find it personally relevant.

Other sociodemographic and psychological variables, like education, income, depression, anxiety, and self-efficacy, also have significant influences on self-care practices [2,8]. This study focused on the sociodemographic, clinical, behavioral, knowledge-based, and psychological factors that were directly measured. This study is based on the Middle-Range Theory of Self-Care of Chronic Illness and the Biopsychosocial Model, which offer a framework that allows one to understand HF self-care from several angles [9,10]. This theoretical approach offers a more comprehensive understanding of the processes that shape the self-care behaviors in HF by means of cognitive, affective, clinical, and socio-psychological factors. In accordance with the Middle-Range Theory, proper self-care maintenance, symptom monitoring, and self-care management depend on the patient’s knowledge, experience, functioning capacity, and psychological condition. On the other hand, the Biopsychosocial Model presents heart failure self-care as a multi-dimensional construct that consists of biological, psychological, and socio-environmental influences (such as severity of the disease, comorbidity, distress, anxiety, motivation, education, income, marital status, and support). Therefore, the combined use of the framework supports understanding the nature of heart failure self-care as a multi-dimensional process that involves clinical, psychosocial, and sociodemographic characteristics. In this study, the Middle-Range Theory informed the inclusion of HF knowledge, functional status (NYHA class, LVEF phenotype), and psychological condition (HADS anxiety and depression) as factors expected to relate to self-care maintenance, symptom perception, and self-care management, while the Biopsychosocial Model informed the inclusion of sociodemographic and socio-environmental factors (age, sex, education, income, work status, residence, marital status) and clinical/behavioral factors (BMI, smoking, physical activity, sleeping difficulty, HF duration, prior HF admission). Self-care confidence was treated as a separate outcome associated with the same set of factors rather than as a mediator formally tested in this cross-sectional design. We hypothesized that higher HF knowledge and lower psychological symptom burden would be associated with higher scores on all three behavioral scales and on the separate Confidence outcome and that sociodemographic and clinical factors would show scale-specific associations; these relationships were treated as associational, not causal, given the cross-sectional design.

Evidence on HF self-care from Jordan specifically remains limited; most available cross-sectional data come from neighboring Middle Eastern and broader regional settings, including Saudi Arabia [7] and, more broadly, situational-theory-based work from Korea [6], which similarly identified knowledge and psychosocial factors as correlates of self-care but did not separate the Symptom Perception scale introduced in SCHFI v7.2 or examine Self-Care Confidence as a distinct outcome. This current study helps to bridge an important research gap by focusing on how various social, demographic, clinical, behavioral, knowledge-based, and psychosocial factors are related to self-care maintenance, symptom perception, and self-care management, with self-care confidence examined as a separate outcome, in HF patients, especially from a Middle Eastern perspective. The primary objective of this study was to examine the cross-sectional associations between sociodemographic, clinical, behavioral, knowledge-based, and psychological factors and self-care maintenance, symptom perception, and self-care management in adults with HF in Jordan, with self-care confidence examined as a separate outcome.

## 2. Methods

### 2.1. Study Design and Setting

The current study was based on a cross-sectional survey design. This study was conducted at two tertiary-care hospitals in northern Jordan—King Abdullah University Hospital and Princess Basma Hospital—both of which provide specialized outpatient heart failure (HF) clinics. These clinics operate with comparable structures, multidisciplinary teams, and standardized care pathways for HF management, including patient education, routine follow-up, and guideline-directed medical therapy. Both hospitals are tertiary-care teaching institutions in the same referral region of northern Jordan and are believed, based on their shared institutional accreditation and standardized national HF care pathways, to serve a broadly similar catchment population with comparable sociodemographic and cultural characteristics; because a site identifier was not retained in the analytic dataset, this similarity could not be empirically verified against the study data, and possible between-site differences in population characteristics or care delivery cannot be ruled out.

### 2.2. Ethical Considerations

This study was conducted in accordance with the ethical principles outlined in the Declaration of Helsinki. Ethical clearance was obtained from the Institutional Review Boards of King Abdullah University Hospital and Princess Basma Hospital (Approval No. Apr2025/181-21) before participant recruitment and data collection commenced. Institutional authorization was also secured to facilitate access to the outpatient heart failure clinics and relevant medical records. Prior to enrollment, all eligible participants were provided with clear information regarding the study objectives, procedures, potential considerations, and their rights as research participants, after which written informed consent was obtained. Participation was entirely voluntary, and participants were informed of their right to decline participation or withdraw without any effect on their clinical care. To protect privacy and confidentiality, each participant was assigned a unique study code, with identifying information separated from research data. All study materials and electronic datasets were stored securely and were accessible only to authorized members of the research team.

### 2.3. Population and Sample

Data collection was performed on a consecutive series of patients with HF who regularly attended HF clinics in two different hospitals in northern Jordan. The current study was conducted between May 2025 and November 2025.

Eligible participants included adults aged 18 years and above with a confirmed diagnosis of HF. The criteria for diagnosis included echocardiogram assessment, ejection fraction, New York Heart Association classification, and clinical manifestations of HF. HF diagnosis was confirmed by the treating cardiologist at each site using this combination of echocardiographic LVEF, NYHA classification, and clinical presentation. For participants with HFmrEF and HFpEF, this clinical-presentation criterion required objective evidence of a structural or functional cardiac abnormality consistent with HF (e.g., elevated natriuretic peptides, left atrial enlargement, or echocardiographic evidence of diastolic dysfunction) in addition to LVEF and symptoms, consistent with contemporary universal HF definitions; this standard was intended to be applied uniformly by cardiologists at both sites, although no separate site-level audit of diagnostic-criteria concordance was conducted, which is acknowledged as a limitation. Participants with HFpEF, HFmrEF, and HFrEF were all eligible once this confirmation was documented; a single, most recent LVEF value from the medical record was used to assign phenotype, and change in phenotype over time (e.g., HF with improved ejection fraction) was not separately tracked. Illiterate participants were also eligible if they had a companion to assist them in the survey. For these participants, the companion read each item aloud and could clarify wording on request, while the participant provided their own responses, which were recorded by the data collector; the number of participants receiving this assistance was not separately logged in the analytic dataset, and the potential for reduced privacy and social-desirability bias in companion-assisted completion, particularly for the HADS, is acknowledged in the Limitations. Excluded participants included those unable to complete the survey because of severe cognitive impairment. Severe cognitive impairment and psychosis were identified from documented clinical diagnoses in the medical record rather than a study-administered cognitive-screening instrument. Individuals with cognitive impairments such as dementia and psychosis, those with New York Heart Association classification IV, and those with acute or decompensated heart failure requiring emergency stabilization, severe chest infections, or other infections that affect quality of life were excluded. Patients with severe comorbid chronic illnesses such as chronic obstructive pulmonary disease, end-stage renal disease, chronic liver disease, and cerebrovascular accidents were also excluded. NYHA class IV and these comorbidities were excluded to limit confounding from acute illness burden and to support reliable completion of self-report measures; this exclusion narrows the study population relative to the full spectrum of HF severity and comorbidity, which is addressed further in the Limitations.

### 2.4. Sample Size and Power

Sample size was determined using a priori omnibus fixed-model multiple linear regression calculation based on 24 predictor degrees of freedom. Assuming Cohen’s f^2^ = 0.15, a two-sided α = 0.05, and 90% power, the minimum required sample was 206 participants; the analytical sample comprised 516 participants. Because association-rule mining does not have an equivalent conventional power formula, its data adequacy is addressed separately. The analysis was limited to 13 antecedent variables and a maximum of three antecedents per rule. Candidate profiles required at least 40 participants, at least 26 participants satisfying both the profile and outcome, support ≥ 5%, confidence ≥ 85%, lift ≥ 1.10, and a prevalence difference ≥ 10 percentage points. Multi-antecedent rules were required to improve lift by at least 0.05 over the strongest available proper subset. Retained profiles also required a fifth-percentile lift > 1.10 across 500 repetitions after random deletion of 20% of participants and a continuous SCHFI mean at least 5 points lower in the profile group. These criteria avoided sparse profiles while retaining clinically interpretable combinations in the sample of 516 participants.

### 2.5. Participant Recruitment

**Figure 1:**
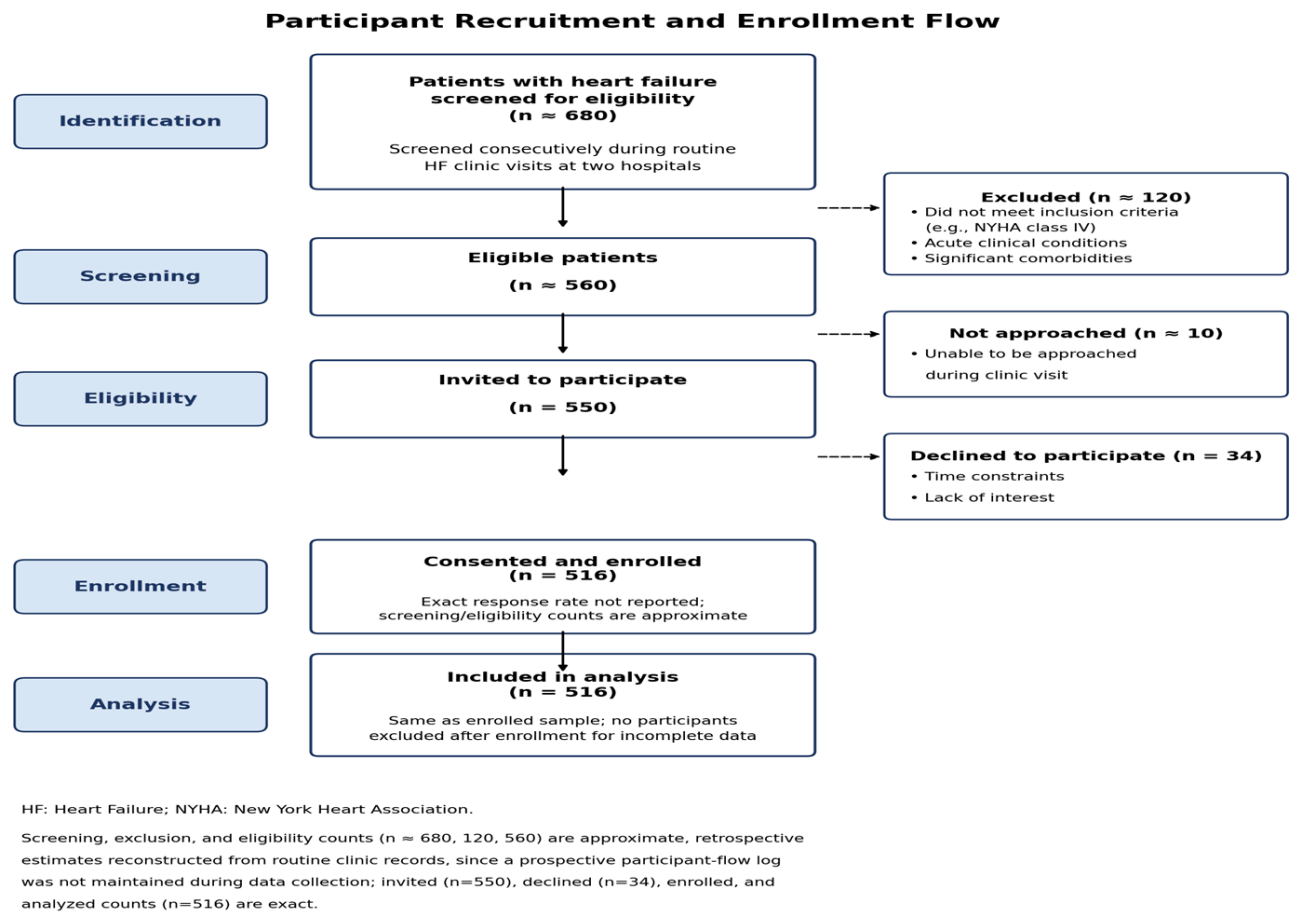
Participant recruitment, eligibility screening, and enrollment flow diagram.

Participant recruitment was carried out using the same procedures at both hospitals. Eligible patients were consecutively screened during routine HF clinic visits based on uniform inclusion and exclusion criteria. Data collection and study protocols were standardized across both sites. Because a prospective participant-flow log was not maintained, the numbers of patients screened and excluded and the eligible retrospective estimates were reconstructed from routine clinic attendance and eligibility review rather than a documented screening record, and a site-level breakdown of these stages is not available because a site identifier was not retained. In total, approximately 680 patients with heart failure were screened for eligibility across the two hospitals (Figure 1). Of these, about 120 were excluded for not meeting the inclusion criteria, including those with advanced heart failure (NYHA class IV), acute clinical conditions, or significant comorbidities, leaving approximately 560 patients eligible. Of these, 550 were approached and invited to participate, and 34 declined, mainly citing time constraints or lack of interest. In total, 516 patients gave written informed consent and were enrolled; this enrolled number is exact, and it is also the analytic sample, since all 516 had complete data for the three behavioral scales, the Confidence scale, and the covariate set, and no participant was excluded after enrollment for incomplete data. Because the screening and eligibility counts are approximate, an exact response rate is not reported; the proportion of approached patients who enrolled was approximately 94%.

### 2.6. Data Collection

This study was conducted in accordance with the principles of the Declaration of Helsinki and received ethical approval from the Institutional Review Boards of King Abdullah University Hospital and Princess Basma Hospital (Approval ID: Apr2025/181-21, date of approval: 21 April 2025), the same single approval number covering both sites. The study was conducted between May 2025 and November 2025 in two tertiary hospitals located in northern Jordan. Both sites were tertiary-care teaching hospitals with dedicated outpatient HF clinics providing standard multidisciplinary HF follow-up care; recruitment and questionnaire-administration procedures were intended to be identical at both sites. Written informed consent was obtained from every participant before any study procedure; for illiterate participants, the accompanying companion read the consent information aloud and the participant provided consent personally, with the companion available as an impartial witness to the consent process. The data collection process began after receiving ethical clearance and authorization from the clinic. Patients who were eligible for the study were screened against the stated inclusion and exclusion criteria by the cardiology doctor and referred to nurses for recruitment in the study, so the cardiologist confirmed protocol eligibility rather than exercising independent discretion over which eligible patients were approached; nurses then approached consecutive eligible clinic attendees until the recruitment target was reached. Patients who showed interest in the research were contacted and made aware of the aim and methodological processes involved in the study, after which they participated in the survey. Questionnaires were interviewer-administered on paper by trained study nurses in a private area of the HF clinic; the same nurses extracted the clinical and medication data from the medical record and linked it to each participant’s questionnaire responses using a study identification number. Whether the administering nurse was also a member of the participant’s usual HF care team was not systematically documented, which is acknowledged as a possible source of social-desirability bias in the Limitations. A formal participant-flow record (numbers assessed, eligible, approached, declining, or excluded before reaching the target of 516) was not retained during data collection; this is acknowledged as a limitation of the recruitment reporting. Patients responded to four questionnaires that took about 50 min to complete, while help from family and friends was offered to those patients who were illiterate. The mode, extent, and privacy of this assistance are described above under Population and Sample.

### 2.7. Instruments

The data collection tools were four in number. The tools were a form for collecting information on sociodemographic, anthropometric, and clinical data; the Hospital Anxiety and Depression Scale (HADS); the Atlanta Heart Failure Knowledge Test (AHFKT); and the Self-Care of Heart Failure Index (SCHFI), which could be completed by the respondents or by collecting data from medical records. The Short Form-36 Health Survey was not analyzed and is not reported as an outcome in this manuscript; the sample size calculation below is based on the reported SCHFI outcomes rather than health-related quality of life.

### 2.8. Sociodemographic, Anthropometric, and Clinical Data Form

The study-specific data form was created to collect information on the sociodemographic and clinical data of the participants. The sociodemographic information included age, sex, educational level, BMI, marital status, employment status, place of residence, living arrangements, monthly income, smoking status, physical activity, and health insurance status. The clinical information was obtained from patients’ medical records, such as physical activity, New York Heart Association (NYHA) classification, presence of comorbid conditions, medication, self-monitoring, LVEF, history of hypertension and diabetes mellitus, and time since diagnosis of HF. Physical activity was collected by self-report on the sociodemographic form and cross-checked against the medical record where documented, and it was dichotomized as active versus not physically active based on the participant’s current regular engagement in physical activity. Current smoking (yes/no) and sleeping difficulty (yes/no) were both self-reported at enrollment. HF duration was categorized from the medical record as diagnosed at this visit, less than 1 year, or 1 year or more since initial HF diagnosis; “diagnosed at this visit” denotes participants with no prior recorded HF diagnosis in the available medical record before the study encounter (n = 87, 16.9% of the sample) rather than a claim that HF onset was necessarily new. This group most plausibly comprises a mix of patients referred to the HF clinic with suspected HF whose diagnosis was confirmed at the study visit and patients with pre-existing but previously undocumented HF; these two scenarios could not be distinguished from the available records, and this heterogeneity is acknowledged as a limitation. This category was retained as the regression reference group because it had an unambiguous operational definition (absence of a prior record) rather than a recalled duration, with the other two categories representing increasing exposure to HF education and self-care experience relative to this reference group; coefficients for HF duration are interpreted cautiously in the Discussion in light of this heterogeneity. Monthly household income was recorded as a continuous value in Jordanian dinars (JD) and re-expressed per 100 JD in the regression models. Prior HF admission was coded yes/no from the medical record and self-reported as any hospitalization for HF decompensation before enrollment; the number of previous admissions among those with a positive history is reported.

Ejection fraction was also considered an important measure of heart efficiency in pumping blood. It was also categorized to determine different types of heart failure, such as heart failure with reduced ejection fraction if LVEF was 40% or lower, heart failure with mildly reduced ejection fraction if LVEF was 41% to 49%, and heart failure with preserved ejection fraction if LVEF was 50% or higher. This classification followed the standard categorization used in contemporary HF guidelines (HFrEF, HFmrEF, HFpEF), applied consistently at both study sites. A single, most recent LVEF value from the medical record was used for phenotype assignment; whether that value reflected an improvement relative to an earlier, lower LVEF (i.e., HF with improved ejection fraction) was not separately tracked. NYHA class was assigned by the treating cardiologist at the study visit and was intended to reflect the patient’s clinical status at the time self-care measures were completed; the interval between the most recent LVEF assessment and study enrollment was not uniformly recorded, which is acknowledged as a limitation. The NYHA classification was also considered to measure the impact of symptoms of heart failure on physical activity, such as walking, exercise, and other physical activities. The sociodemographic, anthropometric, and clinical data form was developed specifically for this study; its translation and back-translation procedure is described here, and the corresponding procedures for the HADS, AHFKT, and SCHFI are described separately for each instrument below, since these instruments differed in whether an existing validated Arabic version was used or a new translation was undertaken for this study.

The BMI was also recorded, and a continuous measure was obtained using a standard formula: weight divided by height squared. According to the literature, BMI was categorized as underweight if less than 18.5, normal weight if 18.5 to 24.9, overweight if 25 to 29.9, mild obesity if 30 to 34.9, and morbid obesity if 35 or higher. These categories correspond to the standard WHO classification: Class I obesity (30.0–34.9), Class II obesity (35.0–39.9), and Class III obesity (≥40.0); the terms “mild” and “morbid” obesity are non-standard and are replaced by these WHO class labels. Table 1 reports BMI as a continuous median (IQR) rather than by these categories.

### 2.9. Hospital Anxiety and Depression Scale (HADS)

The Hospital Anxiety and Depression Scale (HADS) was used in measuring anxiety and depression while minimizing somatic symptoms. This scale was developed by Zigmond and Snaith [11]. It has 14 items in total, 7 of which measure anxiety and 7 of which measure depression. Each item is rated on a 4-point scale ranging from 0 to 3. The range of possible results is 0–21 for each subscale. A high score means a high level and frequency of symptoms. The results can be interpreted as follows: normal (0–7), mild (8–10), moderate (11–14), and severe (15–21). Previous research has shown acceptable reliability results for the Arabic version of the scale; values of 0.78 for anxiety and 0.87 for depression were obtained by using Cronbach’s alpha. Unlike the AHFKT, the HADS was administered using this pre-existing, previously validated Arabic translation rather than a translation developed for this study; the reliability values above are drawn from that prior Arabic validation work, and no new translation or cultural adaptation of the HADS was undertaken here. The Arabic version of the scale is used in this study. The two seven-item subscale scores were calculated separately and entered simultaneously in the multivariable models.

### 2.10. Atlanta Heart Failure Knowledge Test (AHFKT)

The AHFKT has been developed to measure the level of patient knowledge regarding heart failure, its management, and self-care. Version 2 consists of 30 items that cover the heart failure disease process, diet and nutrition, sodium and fluid intake, medications, symptoms, weight monitoring, and physical activity. The instrument was first published in 2009, used in several previous studies, and reevaluated by Butts, et al. [12]. For each correct answer, the patient scores one point, while incorrect answers or skipped ones are awarded zero. The total scores range from 0 to 30. The scores can also be expressed in percentages. Although some of the questions have more than one possible answer, the scoring system is simple. It is a dichotomous scale for the evaluation of patient knowledge. The psychometric properties of the AHFKT are good. These figures are the original-instrument psychometric properties reported by Butts et al. [12] for the English-language AHFKT (content validity indices for clarity and relevance of 0.81 and 0.92, an overall content validity index of 0.96, and Cronbach’s alpha of 0.87); they describe the original English instrument and are distinct from the internal-consistency coefficient of 0.678 observed for the Arabic-translated version in this sample, reported in the Results and interpreted there as moderate rather than as evidence of full validation. Since there was no available translation of the AHFKT in Arabic, the AHFKT was translated into Arabic for this study. Initially, a bilingual nursing expert with advanced U.S. training translated the instrument from English to Arabic. Then, a second translator translated the instrument from Arabic into English. The translation team then compared and reconciled the two versions. The translation of the AHFKT into Arabic was then tested on 10 patients with HF (5 males and 5 females) at the cardiac clinic. The patients found the instrument clear, easy to understand, and culturally appropriate. This procedure evaluated clarity and face acceptability rather than full cross-cultural adaptation; a separate formal cognitive-interviewing phase, calculation of an Arabic-specific content validity index, and psychometric validation (e.g., confirmatory factor analysis, test–retest reliability) were not conducted. Accordingly, the psychometric properties reported above for the original English AHFKT should not be assumed to apply to the Arabic version used here, and validity claims for the Arabic AHFKT are limited to the modest internal-consistency evidence (KR-20 = 0.678) reported in the Results.

### 2.11. Self-Care of Heart Failure Index (SCHFI), Version 7.2

The Self-Care of Heart Failure Index (SCHFI), version 7.2, was used to assess three behavioral scales: Maintenance (10 items), Symptom Perception (11 items), and Management (8 items), together with a separate 10-item Confidence scale. The present analyses used the three behavioral scales. Each scale was scored separately from 0 to 100 using [(observed item sum − minimum possible sum for the observed items)/(maximum possible sum for the observed items − minimum possible sum for the observed items)] × 100. A scale score was calculated when at least 50% of its items had valid responses. For Symptom Perception items B10 and B11, responses coded −1 (“not applicable”) were excluded from the participant-specific numerator and denominator; valid responses of 0 on B10, B11, and Management item C8 were retained. All 516 participants met the scoring rule for each behavioral scale, and no scale-score imputation was used. Higher scores indicate better self-care, and a score of 70 has commonly been used as a reference threshold [8,13]. The separate 10-item Confidence score was calculated on the same 0–100 metric from responses scored 1–5; all 516 participants had valid responses to all 10 items. Confidence was analyzed separately and was not treated as a fourth behavioral scale. The officially available Arabic self-care measures resource corresponds to SCHFI v6.2, a 22-item version that does not include the separate Symptom Perception scale introduced in v7.2; because this study used the v7.2 structure, the v7.2 items were translated and adapted into Arabic for this study following the same forward-translation, back-translation, expert-reconciliation, and 10-patient pilot-testing procedure described above for the AHFKT rather than administering the existing Arabic v6.2. As with the AHFKT, this constitutes evidence of clarity and face acceptability rather than a full cross-cultural validation of the Arabic SCHFI v7.2, which is acknowledged as a measurement limitation.

### 2.12. Statistical Analysis

All analyses were conducted using R 4.5.2 (R Foundation for Statistical Computing). Continuous variables were summarized as medians with interquartile ranges (IQR), and categorical variables were summarized as frequencies with percentages. Internal consistency was assessed using Cronbach’s alpha. Bivariate comparisons of SCHFI behavioral-scale scores across New York Heart Association (NYHA) classes and left ventricular ejection fraction (LVEF) phenotypes were evaluated using Kruskal–Wallis tests. A significant omnibus comparison was followed by Dunn pairwise tests with Holm adjustment within that comparison. Separate ordinary least-squares regression models were fitted for Maintenance, Symptom Perception, and Management to estimate adjusted mean-score differences. The covariates represented the study’s planned sociodemographic, clinical, behavioral, knowledge-related, and psychological domains. The same simultaneous-entry adjustment set was used across the three related behavioral outcomes to maintain comparability: age, sex, BMI, NYHA class, LVEF phenotype, education, monthly income, work status, residence, marital status, current smoking, physical activity, sleeping difficulty, HF duration, prior HF admission, HF knowledge, HADS anxiety, and HADS depression. Monthly income was expressed per 100 Jordanian dinars (JD), HF knowledge per 10 percentage points, and the two HADS subscales were entered simultaneously. Unstandardized coefficients (B), standardized coefficients (β), heteroscedasticity-consistent robust standard error type 3 (HC3) 95% confidence intervals, and *p*-values were reported, together with HC3 overall omnibus tests. Model checks included floor and ceiling frequencies, residual and influence plots, Breusch–Pagan and RESET tests, Shapiro–Wilk residual checks, externally studentized residuals, Cook’s distance, leverage, adjusted generalized variance inflation factors, and HC3 joint tests of standardized quadratic terms for the six continuous predictors. A fourth model used Self-Care Confidence as a separate outcome with the same simultaneous-entry covariates, HC3 inference, diagnostic criteria, and continuous-predictor nonlinearity assessment. The NYHA and LVEF bivariate comparisons were also performed for the separate Confidence scale. All tests were two-sided, and *p* < 0.05 was considered statistically significant.

To assess whether the findings were sensitive to overlapping measurement content, outcome-specific models were fitted using the same analytical sample and HC3 procedures. HF knowledge was omitted from all three models because the AHFKT includes knowledge items concerning symptoms, monitoring, and behaviors represented in the SCHFI scales. Physical activity was additionally omitted from the Maintenance model because exercise is assessed within that scale. The Confidence sensitivity model omitted HF knowledge and otherwise retained its primary adjustment set.

Association-rule analysis was conducted to identify combinations of participant characteristics that co-occurred with Maintenance, Symptom Perception, or Management scores < 70 [14,15]. Thirteen antecedent variables were selected to represent the five analytic domains while retaining interpretable categories and sufficient participant counts: age, sex, BMI, NYHA class, LVEF category, education, current smoking, sleeping difficulty, prior HF admission, comorbidity burden, HF knowledge, HADS anxiety, and HADS depression. Individual questionnaire items, duplicate representations of the same construct, and physical activity were excluded; physical activity directly overlaps with the Maintenance scale. Rules were restricted to a maximum of three antecedents and retained when the antecedent profile included at least 40 participants, at least 26 participants satisfied both antecedent and outcome, support was ≥5%, confidence was ≥85%, lift was ≥1.10, and the prevalence difference between participants inside and outside the profile was ≥10 percentage points. These retention thresholds, the antecedent list, and the SCHFI < 70 consequent were specified before the association-rule analysis was run and were not adjusted after inspecting the resulting rules. Multi-antecedent rules were required to improve lift by at least 0.05 over the strongest available proper subset. Selected metrics were recomputed in 500 repetitions after random deletion of 20% of participants; the practical shortlist required a 5th-percentile lift > 1.10 and a continuous SCHFI mean at least 5 points lower in the profile group. With 516 participants, these count thresholds avoided sparse profiles while retaining clinically interpretable combinations. The results were treated as descriptive co-occurrence profiles rather than interactions, causal effects, or predictive models. The separate Confidence score < 70 was included as an additional rule consequent under the same criteria.

The present study utilizes the same patient cohort from our previous investigation on health-related quality of life [16]. However, this analysis diverges in scope, focusing exclusively on distinct clinical endpoints: specifically, heart failure self-care behaviors and their underlying determinants.

## 3. Results

The sample size comprised 516 respondents. All 516 participants had complete item responses for the three behavioral scales, complete Confidence-item responses, and complete covariate data and were included in each model. The median age was 60 years (Q1–Q3: 53.0–64.5), and the vast majority of respondents were males (81%) and married (93%). Almost half (44%) of the respondents had higher education, while 40% had secondary education. The median income per month was 400 JD (Q1–Q3: 200–500). With regard to occupation status, 41%, 31%, and 28% of the respondents were retired, working, and not working, respectively (Table 1). The item-level Symptom Perception estimate used the 427 participants with complete applicable responses.

Cronbach’s alpha was 0.796 for HADS anxiety, 0.716 for HADS depression, 0.803 for Maintenance, 0.840 for Symptom Perception, and 0.769 for Management. Cronbach’s alpha for the 10-item Confidence scale was 0.946. The AHFKT knowledge items demonstrated moderate internal consistency (KR-20 = 0.678).

**Figure 2:**
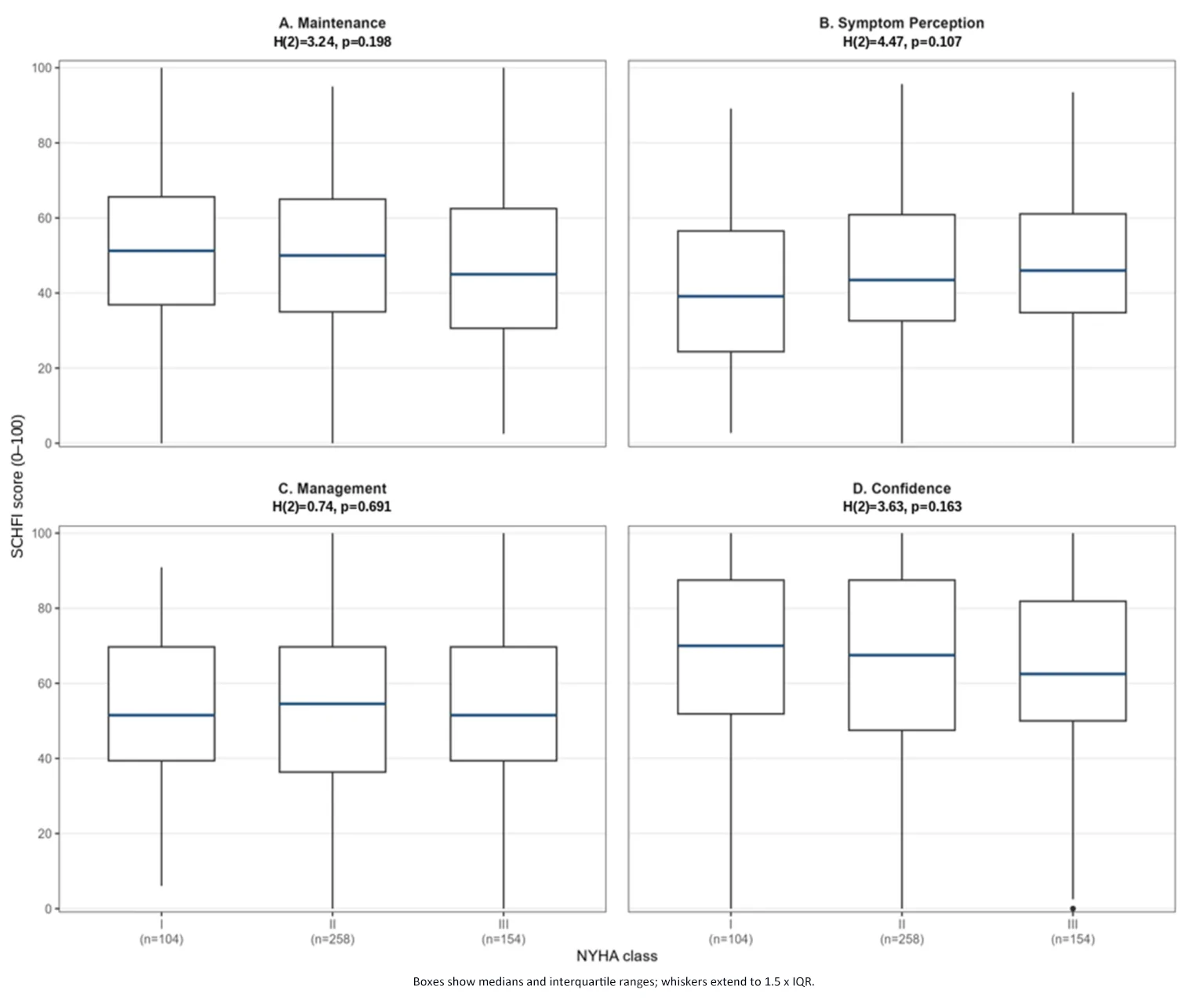
SCHFI Maintenance, Symptom Perception, and Management scores by NYHA class. The fourth panel presents the separate Self-Care Confidence scale (bivariate *p*-values shown in panel headers).

Group-specific distributions are reported in Table 2 and displayed in Figure 2. Maintenance did not differ across NYHA classes, H(2) = 3.24, *p* = 0.198; neither did Symptom Perception, H(2) = 4.47, *p* = 0.107, or Management, H(2) = 0.74, *p* = 0.691. Self-Care Confidence yielded H(2) = 3.63, *p* = 0.163 across NYHA classes.

**Figure 3:**
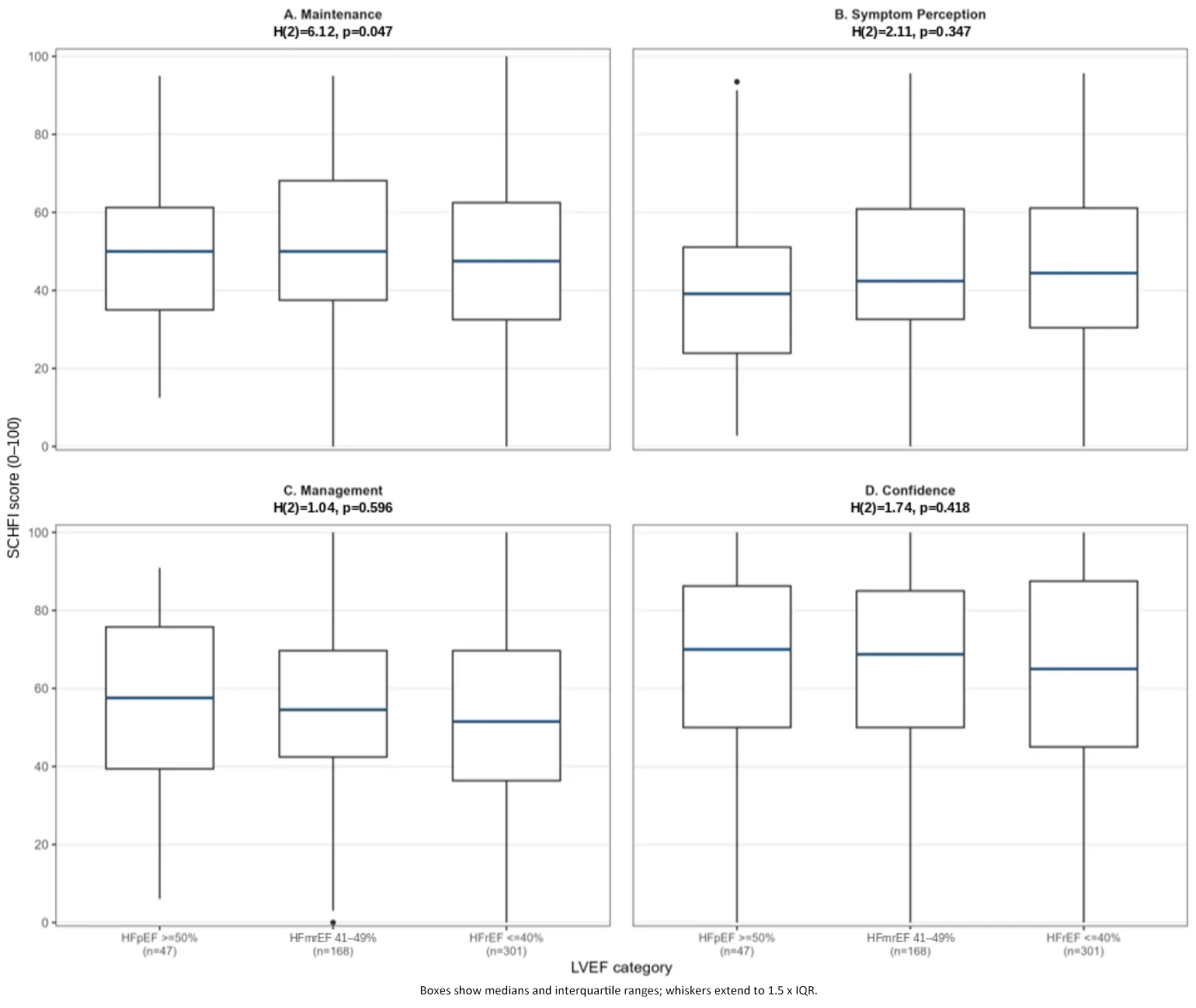
SCHFI Maintenance, Symptom Perception, and Management scores by LVEF category. The fourth panel presents the separate Self-Care Confidence scale (bivariate *p*-values shown in panel headers).

Maintenance differed across LVEF phenotypes, H(2) = 6.12, *p* = 0.047 (Table 2 and Figure 3). Dunn comparisons with Holm adjustment showed a difference between HFmrEF and HFrEF (adjusted *p* = 0.048); HFpEF versus HFmrEF (adjusted *p* = 0.705) and HFpEF versus HFrEF (adjusted *p* = 0.560) were not significant. Symptom Perception, H(2) = 2.11, *p* = 0.347, and Management, H(2) = 1.04, *p* = 0.596, did not differ across LVEF phenotypes. LVEF phenotype was not independently associated with any behavioral scale in the adjusted models. Self-Care Confidence yielded H(2) = 1.74, *p* = 0.418 across LVEF phenotypes.

The median SCHFI score was 50.00 (IQR = 35.00–65.00) for Maintenance, 43.48 (IQR = 30.43–60.87) for Symptom Perception, and 51.52 (IQR = 39.39–69.70) for Management (Table 2). Floor frequencies were 0.58%, 0.58%, and 0.39%, respectively; ceiling frequencies were 0.39%, 0%, and 0.39%. The separate Self-Care Confidence score had a median of 67.50 (IQR = 50.00–86.25), an observed range of 0.00–100.00, a floor frequency of 7 (1.36%), and a ceiling frequency of 36 (6.98%).

In the multivariable Maintenance model, elementary education (B = −10.02, 95% CI [−16.34, −3.70], *p* = 0.002) and secondary education (B = −6.39, 95% CI [−10.91, −1.87], *p* = 0.006) were inversely associated with Maintenance relative to higher education (Table 3). A 10-percentage-point increase in HF knowledge was associated with a 2.02-point higher score (95% CI [0.50, 3.54], *p* = 0.009), whereas each one-point increase in HADS depression was associated with a 0.94-point lower score (95% CI [−1.57, −0.31], *p* = 0.004). The model had R^2^ = 0.119 and adjusted R^2^ = 0.076.

In the Symptom Perception model, current smoking (B = −6.69, 95% CI [−10.78, −2.60], *p* = 0.001) and no prior HF admission (B = −4.32, 95% CI [−8.21, −0.42], *p* = 0.030) were inversely associated with the score. A 10-percentage-point increase in HF knowledge was associated with a 2.26-point higher score (95% CI [0.78, 3.73], *p* = 0.003). With anxiety, depression, and the remaining covariates entered simultaneously, HADS anxiety was positively associated with Symptom Perception (B = 1.37, 95% CI [0.74, 2.00], *p* < 0.001), whereas HADS depression was inversely associated (B = −1.47, 95% CI [−2.10, −0.83], *p* < 0.001). The NYHA class III coefficient was at the reporting boundary (B = 5.79, 95% CI [0.00, 11.58], *p* = 0.050). The model had R^2^ = 0.148 and adjusted R^2^ = 0.107.

In the Management model, a 10-percentage-point increase in HF knowledge was associated with a 3.81-point higher score (95% CI [2.32, 5.30], *p* < 0.001). With anxiety, depression, and the remaining covariates entered simultaneously, HADS anxiety was positively associated with Management (B = 0.92, 95% CI [0.33, 1.51], *p* = 0.002), whereas HADS depression was inversely associated (B = −1.09, 95% CI [−1.70, −0.48], *p* < 0.001). The model had R^2^ = 0.130 and adjusted R^2^ = 0.088.

In the Self-Care Confidence model, the statistically significant adjusted associations were HF knowledge, per 10 percentage points (B = 3.58, 95% CI [1.88, 5.28], *p* < 0.001); HADS anxiety, 0–21 (B = 0.75, 95% CI [0.05, 1.44], *p* = 0.036); and HADS depression, 0–21 (B = −2.22, 95% CI [−2.92, −1.52], *p* < 0.001). The model had R^2^ = 0.168 and adjusted R^2^ = 0.127.

The HC3 overall omnibus tests were significant for Maintenance, F(24, 491) = 2.96, *p* < 0.001, Symptom Perception, F(24, 491) = 3.42, *p* < 0.001, and Management, F(24, 491) = 3.24, *p* < 0.001. Some residual and specification tests indicated modest departures: the Breusch–Pagan test was significant for Maintenance and Management, Shapiro–Wilk tests were significant for Maintenance and Symptom Perception, and the Maintenance RESET was *p* = 0.045. However, the HC3 joint tests of quadratic terms were not significant for Maintenance (*p* = 0.215), Symptom Perception (*p* = 0.991), or Management (*p* = 0.228). The maximum adjusted generalized variance inflation factor was 1.45; no observation had Cook’s distance > 1; one Maintenance observation had an externally studentized residual exceeding |3|; and nine observations exceeded the 2p/N leverage threshold in each model. Full numerical results and diagnostic plots are provided in Supplementary File S1. For Self-Care Confidence, the HC3 omnibus test was F(24, 491) = 4.93, *p* < 0.001; the Breusch–Pagan *p*-value was 0.071, the Shapiro–Wilk *p*-value was <0.001, RESET *p* = 0.596, and the joint quadratic-term *p*-value was 0.331. The maximum adjusted GVIF was 1.448; 0 observations had Cook’s distance > 1, and 9 exceeded the 2p/N leverage threshold.

The sensitivity models remained statistically significant for Maintenance, F(22, 493) = 2.70, *p* < 0.001; Symptom Perception, F(23, 492) = 3.14, *p* < 0.001; and Management, F(23, 492) = 1.77, *p* = 0.015. Adjusted R^2^ values were 0.061, 0.089, and 0.037, respectively. No predictor retained in these models gained or lost statistical significance relative to the corresponding primary model, with one minor exception: the NYHA class III coefficient in the Symptom Perception model crossed the *p* < 0.05 threshold, from *p* = 0.050 in the primary model to *p* = 0.046 in the sensitivity model; this small shift does not materially alter the pattern of findings. The Self-Care Confidence sensitivity model had adjusted R^2^ = 0.096 and HC3 F(23, 492) = 4.06, *p* < 0.001. In the Confidence sensitivity model excluding HF knowledge, HADS anxiety, 0–21 (B = 0.70, 95% CI [−0.01, 1.41], *p* = 0.053), no longer met the *p* < 0.05 threshold. No other retained Confidence predictor crossed that threshold (Supplementary Tables S9 and S10).

Maintenance, Symptom Perception, and Management scores < 70 occurred in 410 (79.5%), 443 (85.9%), and 406 (78.7%) participants, respectively. Of 9516 enumerated rules, 245 met the initial criteria, 73 remained after redundancy pruning, 15 underwent case-deletion analysis, and 10 met the practical shortlist criteria (Table 4). The highest lift was observed for male sex, sleeping difficulty, and HF knowledge from 50% to <60% with Management < 70 (50/51 participants; confidence = 98.0%; lift = 1.246; prevalence difference = 21.5 percentage points). For Maintenance, male sex, no prior HF admission, and HADS depression 11–21 yielded confidence = 95.2% and lift = 1.199. For Symptom Perception, LVEF > 40%, current smoking, and no prior HF admission yielded confidence = 98.1% and lift = 1.143. For the separate Confidence outcome, 267 participants (51.7%) had scores < 70, and no profiles met the same final criteria. The Confidence analysis enumerated 3172 rules.

## 4. Discussion

The findings from this study reveal substantial variations in self-care behaviors among patients with HF, with median SCHFI scores indicating suboptimal performance across the three behavioral scales. A study performed by Vatmasari, et al. [17] reported that the median maintenance score and symptom perception score suggest that nearly half of the patients struggle with fundamental self-care activities. Although self-care confidence appeared relatively higher, the discrepancy between patients’ perceived ability and their actual performance in self-care maintenance and symptom perception remains an important issue that requires further attention. This finding was consistent with a study conducted by Tian, et al. [18], which emphasized that multiple interrelated factors contribute to poor self-care performance.

The associations reported below are interpreted as cross-sectional and bidirectional rather than as evidence of a single causal direction. For example, greater HF knowledge may facilitate self-care, but sustained engagement in self-care, prior decompensation, and repeated contact with the healthcare system may equally increase a patient’s HF knowledge over time; a similar bidirectionality likely applies to the associations involving psychological symptoms, symptom perception, and self-care confidence. Likewise, NYHA functional class and LVEF phenotype reflect the underlying cardiac condition, comorbidity, and treatment response rather than self-care itself, so the absence of an independent association between disease severity and self-care in the adjusted models should not be read as evidence that severity is irrelevant to self-care, nor should a positive association elsewhere be read as evidence that self-care determines functional class. The adjusted R^2^ values for the four models ranged from 0.076 to 0.127, indicating that the measured covariates explained a limited proportion of the variability in self-care maintenance, symptom perception, self-care management, and self-care confidence; most of this variability remains unexplained by the present model and likely reflects unmeasured cultural, coping, health literacy, and social support factors.

Knowledge of HF was a significant and consistent correlate across all self-care domains. In this study, HF knowledge showed a significant positive relationship with self-care maintenance, symptoms of HF, self-care management, and self-care confidence, with the largest standardized coefficient observed for self-care management (β = 0.237). This is in agreement with D’Souza, et al. [19], who emphasized the need to recognize the role of disease-specific education, where patients with higher knowledge about their condition are able to better recognize symptoms, understand medication regimens, and make lifestyle changes. Likewise, Dessie, et al. [20] showed that educational interventions have a significant effect on self-care adherence for patients with HF. The relationship between knowledge and self-care management observed here is also consistent with a study by Baymot, et al. [21], where they stressed the need for knowledge to help patients respond to exacerbations of symptoms. This association is of particular interest, especially considering that lack of knowledge has been identified as a major barrier to self-care, as indicated by the study by Astuti, et al. [22], which identified depression symptoms and e-Health literacy as major predictors of self-care among patients with heart failure. Moreover, Dessie, et al. [20] indicated that patient education programs have shown success in enhancing patient knowledge and self-care adherence. This supports the theoretical framework developed by Riegel, et al. [9], which highlights the importance of knowledge as a foundational element of self-care. This has also been established by other studies that have shown that increased knowledge of disease can improve adherence, symptoms, and decision-making [23].

Additionally, the level of education was an important sociodemographic correlate, especially for the maintenance of self-care. Specifically, patients with an elementary level of education were found to have lower maintenance scores compared to those with higher levels of education. Along the same lines, Van Driel, et al. [24] proposed that the level of educational attainment may affect the cognitive ability to comprehend self-care instructions, take complex medication regimens, and make lifestyle changes, as health literacy, which is closely associated with the level of education, is considered an important correlate of self-care performance among patients with HF. The educational gradient in maintaining self-care is consistent with Janz and Becker [25], who found that it is important to understand how patients perceive health information to facilitate behavioral change. Furthermore, this is also consistent with the findings of Bekele, et al. [26], who found a positive relationship between higher educational level and self-care adherence, consistent with the direction of the Maintenance finding in this sample. The pattern observed here was not strictly monotonic: relative to higher education, both elementary education (B = −10.02) and secondary education (B = −6.39) were significantly associated with lower Maintenance scores, whereas the no-education category was not significantly different (B = −1.70, *p* = 0.770), likely reflecting its small size (n = 26) and the possibility that companion-assisted questionnaire completion, more common in this subgroup, may have influenced responses. These findings should therefore be interpreted as scale-specific associations rather than as evidence of a strict educational dose–response gradient.

Interestingly, monthly income did not attain statistical significance in the multivariable models despite the trend of negative association with confidence scores. In similarity with other socio-economic studies, Powell-Wiley, et al. [27] observed that the social determinants of health, which include the economic, social, environmental, and psychosocial factors that influence health, have a major role in the development of cardiovascular disease. Additionally, other studies found income levels to be significant factors in health literacy and adherence [20,28].

Women had a higher adjusted Symptom Perception score than men, although this association did not reach statistical significance in the adjusted model (B = 4.84, 95% CI −0.79 to 10.46, *p* = 0.092); this estimate should not be interpreted as evidence that women perceive their HF symptoms more accurately, and the small number of women in this sample (n = 98, 19.0%) limits the precision of this comparison. Sex differences in symptom perception have been discussed as a hypothesis, not an established mechanism, in the broader literature. In relation to this hypothesis, Vatmasari, Putra, Windarwati, and Hany [17] noted that differences in symptom perception between the genders may be due to differences in health awareness, healthcare-seeking behavior, and symptom reporting between the sexes. Prior work has similarly hypothesized that women may report symptoms more consistently and engage more in self-surveillance, although this remains a proposed explanation rather than an established mechanism [17,21]. Behavioral factors like smoking had a negative association with the perception of symptoms, consistent with prior work suggesting that unhealthy behaviors such as smoking are associated with reduced disease awareness and self-management [21,25].

The HADS revealed a notable association between self-care confidence and anxiety and depression. Depression was inversely associated with self-care confidence (B = −2.22, 95% CI −2.92 to −1.52), the largest standardized coefficient (β = −0.373) among the Confidence model predictors, and anxiety was positively associated with confidence (B = 0.75, 95% CI 0.05 to 1.44). This is consistent with evidence that depression and anxiety are associated with reduced motivation, self-efficacy, and adherence [20,29]. Psychological burden is also increasingly seen as a major barrier to the self-care of individuals with HF [19]. Consistent with this, the study by Sooyeon and Hanyi [30] indicated the role of depressive symptoms and disease uncertainty as a mediator between physical symptoms and self-care behavior in older patients with HF. Similarly, Bair, et al. [31] found depression and painful symptoms commonly occur together, which negatively affects the recognition and treatment of depression, with implications for self-care engagement.

NYHA functional classification and LVEF phenotype were not independently associated with any of the three behavioral scale scores in the adjusted models. Because functional class and LVEF phenotype primarily reflect the underlying cardiac condition, comorbidity, and treatment response rather than self-care behavior itself, this null finding does not indicate that disease severity is unrelated to self-care in a broader sense, only that it was not an independent correlate after adjustment for the other covariates in this sample. A related descriptive finding is reported by Matsui, et al. [32], that factors such as poor self-care behavior correlate with physical and social frailty in older patients with HF, indicating that there are other factors that influence self-care performance in this population.

Men accounted for 81% of this sample, a higher proportion than the roughly equal sex distribution reported in some contemporary HF cohorts, although sex distribution varies by geographic region and HF phenotype, with men more often represented in HFrEF and women in HFpEF. This predominance is consistent with referral patterns previously described for tertiary HF clinics in neighboring Middle Eastern settings. It does not appear to be explained by a greater concentration of HFrEF among men in this sample: the LVEF-phenotype distribution was nearly identical between men and women (HFrEF in 58.6% of men versus 57.1% of women; Supplementary Table S8), so the male predominance should not be attributed to a sex difference in phenotype composition. Whether it instead reflects center-specific referral or other geographic factors could not be evaluated because a site identifier was not retained in the analytic dataset. The lower symptom-perception scores associated with no prior HF admission (B = −4.32, 95% CI −8.21 to −0.42) should also be interpreted cautiously: the admission variable was coded as ever/never and does not capture the number, recency, or clinical context of any decompensation, so this association may reflect differences in symptom experience, prior professional contact, or education received at a previous admission rather than a direct effect of admission history itself. Multivariable regression analysis showed some degree of explanation, where the results indicated a minor amount of explained variance in the different self-care dimensions. The current finding is supported by previous research on self-care in heart failure, where the process of self-care behavior is found to involve a number of complicated interactions that transcend conventional demographic and clinical factors. A study performed by Tian, et al. [18] reported that self-care in HF is associated with a number of psychosocial factors, including health beliefs, culture, coping styles, and others. Therefore, the findings indicate the complexity of factors affecting self-care practices and the necessity of identifying more psychological, behavioral, social, and cultural factors involved in such processes.

## 5. Clinical Implications and Recommendations

The results from this study indicate that a multi-dimensional strategy for the management of self-care among patients with heart failure is required. The specific contribution of this study is descriptive: HF knowledge was associated with higher self-care-related scores across Maintenance, Symptom Perception, and Management, and HADS depression was associated with lower self-care confidence, while HADS anxiety was positively associated with confidence, in this cross-sectional sample. These associations may help inform further research and highlight a possible need to evaluate patients’ support needs around knowledge and psychological symptoms; they do not indicate that educational or psychological interventions increase self-care abilities, health-related quality of life, hospital admissions, or mortality because no intervention was tested and the cross-sectional design cannot establish this. Multidisciplinary care and structured patient education are well-established elements of guideline-based HF management, but this study did not evaluate multidisciplinary care, structured educational interventions, or family or caregiver participation; assistance provided by companions in completing the questionnaires does not constitute a measurement of caregiver or family support, and general recommendations regarding family involvement are therefore not presented as findings of this study.

Proper self-care is widely recognized in the broader HF literature as important for preventing clinical decline, emergency care, and unnecessary rehospitalizations through medication adherence, symptom recognition, and prompt help-seeking, although this study did not directly test these downstream outcomes. Prior intervention literature suggests that structured education may help improve self-care practices and, in turn, quality of life and admission rates; the present cross-sectional associations are consistent with a possible role for HF knowledge in self-care but do not themselves demonstrate that educational interventions improve these downstream outcomes among patients with HF.

## 6. Limitations

Although this study has shed important light on factors associated with self-care in heart failure, some limitations should be noted. Like many other studies using a cross-sectional approach, the ability to make causal inferences and understand the longitudinal relationship between the studied factors and the outcome of self-care is restricted. In accordance with this limitation, the limited explanatory power of the models implies that other factors that were not included in the current study, such as cultural factors and coping strategies, and the dynamic nature of self-efficacy, could be important correlates of self-care. It is recommended that in the future, longitudinal designs, as well as the incorporation of other psychological and social factors, should be included, along with the exploration of the mediating and moderating effects of the mechanisms by which knowledge, sociodemographic factors, and psychological well-being relate to self-care behavior. Several additional design and reporting limitations should be considered. Recruitment used consecutive, non-probability sampling with physician-mediated screening for eligibility at two tertiary-hospital HF clinics; a formal participant-flow record was not retained, so selection bias, non-response bias, and possible between-center heterogeneity could not be formally evaluated. The sample was predominantly male (81.0%) and married (93.4%), and NYHA class IV and several common HF comorbidities were excluded, which together limit the transferability of these findings to the full spectrum of HF severity, women, and unmarried patients. Data were collected by self-report and record extraction over approximately 50 min per participant, which may have introduced fatigue effects, social-desirability bias, and, for illiterate participants completing the survey with companion assistance, some reduction in response independence and privacy, particularly for the HADS. The AHFKT was translated for this study and evaluated only for clarity in 10 patients, and the language-version history of the SCHFI required for full v7.2 administration in Arabic could not be independently confirmed against the official validated instrument list; both should be regarded as translated rather than fully cross-culturally validated instruments in this sample, and this is acknowledged as a measurement limitation. These limitations, together with the low explanatory power of the models, the absence of a global multiplicity adjustment, and the lack of temporal direction discussed below, indicate that the findings should be regarded as hypothesis-generating rather than confirmatory. No study protocol or analysis plan was formally preregistered; the regression covariate set was specified a priori based on the conceptual framework described in the Introduction, and the association-rule retention criteria were likewise specified before the analysis was run, but in the absence of a public preregistration, the overall analysis should be regarded as exploratory rather than confirmatory.

Some predictors were conceptually related to SCHFI content, although direct item-content overlap was most evident for HF knowledge and physical activity. Sensitivity analyses excluding HF knowledge from all three models and physical activity from the Maintenance model did not change the statistical significance of any retained predictor. Sleeping difficulty and psychological symptoms were retained because they represent distinct clinical constructs rather than duplicate SCHFI items; nevertheless, some common-method variance may remain because most measures were collected by self-report at the same assessment. The adjusted R^2^ values of 0.076–0.127 indicate that the measured covariates explained a limited proportion of variation in the behavioral scales and Self-Care Confidence. The unmeasured social, cultural, coping, health-literacy, and support factors may also be relevant.

## 7. Conclusions

Based on the results of this study, most participants had scores below 70 on self-care maintenance, symptom perception, and self-care management, suggesting suboptimal self-care in this sample. Self-care confidence scores were somewhat higher on average, but a substantial proportion of participants also scored below this threshold, indicating that higher perceived confidence did not consistently correspond to higher scores on the three behavioral scales. HF knowledge was the most consistent correlate across all three behavioral scales and the separate Confidence outcome, with higher knowledge associated with higher scores throughout, which is consistent with education having a role in HF self-care. HADS depression was associated with lower self-care confidence and, additionally, with lower scores on all three behavioral scales, whereas HADS anxiety was positively associated with self-care confidence and with the Symptom Perception and Management scales; an unexpected direction that should be interpreted cautiously given its sensitivity to model specification. Because the design was cross-sectional and the adjusted R^2^ values were limited (0.076–0.127), these findings should be interpreted as associations rather than as evidence of prediction or causation; the direction of these relationships cannot be established from this design, and longitudinal and intervention studies are needed to determine whether changes in HF knowledge or psychological symptoms lead to improvements in self-care.

## Figures and Tables

**Table 1:** Sociodemographic, clinical, behavioral, knowledge, and psychological characteristics of the study sample.

Characteristic	N = 516
Age, years	60.00 (53.00–64.50)
Sex	
Male	418 (81.0%)
Female	98 (19.0%)
Education	
Higher	226 (43.8%)
No education	26 (5.0%)
Elementary	59 (11.4%)
Secondary	205 (39.7%)
Monthly income, JD	400.00 (200.00–500.00)
Work status	
Working	158 (30.6%)
Retired	212 (41.1%)
Not working	146 (28.3%)
Residence	
Urban	316 (61.2%)
Rural	200 (38.8%)
Marital status	
Married	482 (93.4%)
Unmarried	34 (6.6%)
BMI, kg/m^2^	28.26 (25.45–32.14)
NYHA class	
I	104 (20.2%)
II	258 (50.0%)
III	154 (29.8%)
LVEF phenotype	
HFpEF (LVEF ≥ 50%)	47 (9.1%)
HFmrEF (LVEF 41–49%)	168 (32.6%)
HFrEF (LVEF ≤ 40%)	301 (58.3%)
Current smoking	
Yes	220 (42.6%)
No	296 (57.4%)
Physical activity	
Active	258 (50.0%)
Not active	258 (50.0%)
Sleeping difficulty	
Yes	235 (45.5%)
No	281 (54.5%)
HF duration	
Diagnosed at this visit	87 (16.9%)
<1 year	121 (23.4%)
≥1 year	308 (59.7%)
Prior HF admission	
Yes	269 (52.1%)
No	247 (47.9%)
Number of prior HF admissions among previously admitted participants	3 (1–5)
Comorbidities	
Hypertension	272 (52.7%)
Diabetes mellitus	215 (41.7%)
Coronary artery disease	250 (48.4%)
Hypercholesterolemia	166 (32.2%)
Atrial fibrillation	63 (12.2%)
Comorbidity burden	
0–1 conditions	230 (44.6%)
≥2 conditions	286 (55.4%)
HF medications	
Number of reported HF medication classes	1 (0–2)
Aldosterone antagonist	50 (9.7%)
Digoxin	50 (9.7%)
ACE inhibitor or ARB	51 (9.9%)
Beta-blocker	101 (19.6%)
Loop diuretic	184 (35.7%)
Vasodilator	125 (24.2%)
Calcium-channel blocker	33 (6.4%)
Ivabradine	24 (4.7%)
Valsartan-containing therapy	90 (17.4%)
Did not know medication	232 (45.0%)
HF knowledge, AHFKT percentage correct	53.33 (43.33–60.00)
HADS anxiety, 0–21	8.00 (4.00–11.00)
HADS depression, 0–21	8.00 (5.00–11.00)

Note. Continuous variables are median (IQR); categorical variables are n (%). Medication categories are not mutually exclusive. The number of prior admissions is summarized among the 269 participants reporting a previous HF admission. Comorbidity burden counts the five conditions listed above. Complete data were available for all regression variables.

**Table 2:** SCHFI behavioral-scale and Self-Care Confidence scale distributions in the total sample and across NYHA and LVEF groups.

Panel A. Total-Sample Distributions					
**Scale**	**n**	**Median (IQR)**	**Observed Range**	**Floor, n (%)**	**Ceiling, n (%)**
Maintenance	516	50.00 (35.00–65.00)	0.00–100.00	3 (0.58%)	2 (0.39%)
Symptom Perception	516	43.48 (30.43–60.87)	0.00–95.65	3 (0.58%)	0 (0.00%)
Management	516	51.52 (39.39–69.70)	0.00–100.00	2 (0.39%)	2 (0.39%)
Confidence	516	67.50 (50.00–86.25)	0.00–100.00	7 (1.36%)	36 (6.98%)
**Panel B. Distributions by NYHA class**					
**Scale**	**Group**	**n**	**Median (IQR)**	**H(df)**	***p***
Maintenance	I	104	51.25 (36.25–66.25)	3.24(2)	0.198
Maintenance	II	258	50.00 (35.00–65.00)		
Maintenance	III	154	45.00 (30.00–62.50)		
Symptom Perception	I	104	39.13 (24.39–56.52)	4.47(2)	0.107
Symptom Perception	II	258	43.48 (32.61–60.87)		
Symptom Perception	III	154	46.00 (34.78–61.11)		
Management	I	104	51.52 (39.39–69.70)	0.74(2)	0.691
Management	II	258	54.55 (36.36–69.70)		
Management	III	154	51.52 (39.39–69.70)		
Confidence	I	104	70.00 (51.25–87.50)	3.63(2)	0.163
Confidence	II	258	67.50 (47.50–87.50)		
Confidence	III	154	62.50 (50.00–82.50)		
**Panel C. Distributions by LVEF phenotype**					
**Scale**	**Group**	**n**	**Median (IQR)**	**H(df)**	***p***
Maintenance	HFpEF (≥50%)	47	50.00 (35.00–62.50)	6.12(2)	0.047
Maintenance	HFmrEF (41–49%)	168	50.00 (37.50–68.75)		
Maintenance	HFrEF (≤40%)	301	47.50 (32.50–62.50)		
Symptom Perception	HFpEF (≥50%)	47	39.13 (23.91–52.17)	2.11(2)	0.347
Symptom Perception	HFmrEF (41–49%)	168	42.39 (32.61–60.87)		
Symptom Perception	HFrEF (≤40%)	301	44.44 (30.43–61.11)		
Management	HFpEF (≥50%)	47	57.58 (39.39–75.76)	1.04(2)	0.596
Management	HFmrEF (41–49%)	168	54.55 (42.42–69.70)		
Management	HFrEF (≤40%)	301	51.52 (36.36–69.70)		
Confidence	HFpEF (≥50%)	47	70.00 (50.00–87.50)	1.74(2)	0.418
Confidence	HFmrEF (41–49%)	168	68.75 (50.00–85.00)		
Confidence	HFrEF (≤40%)	301	65.00 (45.00–87.50)		

**Table 3:** Multivariable linear regression models for the three SCHFI behavioral scales. Panel B reports the separate Self-Care Confidence model.

Predictor	Maintenance B [95% CI]; β; *p*	Symptom Perception B [95% CI]; β; *p*	Management B [95% CI]; β; *p*
Age, years	0.10 [−0.13, 0.33] β = 0.047; *p* = 0.371	0.04 [−0.18, 0.25] β = 0.017; *p* = 0.734	0.16 [−0.08, 0.39] β = 0.070; *p* = 0.192
Female (ref: male)	4.17 [−1.44, 9.77] β = 0.079; *p* = 0.145	4.84 [−0.79, 10.46] β = 0.092; *p* = 0.092	3.65 [−2.07, 9.37] β = 0.068; *p* = 0.211
BMI, kg/m^2^	0.05 [−0.33, 0.43] β = 0.014; *p* = 0.794	0.25 [−0.04, 0.54] β = 0.072; *p* = 0.086	0.30 [−0.10, 0.69] β = 0.082; *p* = 0.142
NYHA class II (ref: I)	−0.52 [−5.74, 4.71] β = −0.012; *p* = 0.846	3.47 [−1.44, 8.37] β = 0.084; *p* = 0.165	2.11 [−2.83, 7.05] β = 0.050; *p* = 0.402
NYHA class III (ref: I)	−0.62 [−6.61, 5.36] β = −0.014; *p* = 0.839	5.79 [0.00, 11.58] β = 0.128; *p* = 0.050	4.56 [−1.25, 10.36] β = 0.099; *p* = 0.124
HFmrEF, LVEF 41–49% (ref: HFpEF)	2.32 [−4.63, 9.27] β = 0.052; *p* = 0.512	5.32 [−2.46, 13.10] β = 0.121; *p* = 0.179	1.15 [−5.75, 8.04] β = 0.026; *p* = 0.744
HFrEF, LVEF ≤ 40% (ref: HFpEF)	−1.13 [−7.85, 5.59] β = −0.027; *p* = 0.742	5.10 [−2.53, 12.73] β = 0.122; *p* = 0.190	−1.48 [−8.31, 5.35] β = −0.035; *p* = 0.671
No education (ref: higher)	−1.70 [−13.09, 9.69] β = −0.018; *p* = 0.770	−2.85 [−11.89, 6.19] β = −0.030; *p* = 0.536	1.58 [−9.46, 12.62] β = 0.016; *p* = 0.778
Elementary education (ref: higher)	−10.02 [−16.34, −3.70] β = −0.154; *p* = 0.002	−0.71 [−7.14, 5.72] β = −0.011; *p* = 0.827	−3.67 [−10.61, 3.28] β = −0.055; *p* = 0.300
Secondary education (ref: higher)	−6.39 [−10.91, −1.87] β = −0.151; *p* = 0.006	−0.23 [−4.78, 4.33] β = −0.005; *p* = 0.922	−0.93 [−5.61, 3.76] β = −0.022; *p* = 0.698
Monthly income, per 100 JD	−0.30 [−0.96, 0.37] β = −0.043; *p* = 0.377	−0.10 [−0.79, 0.59] β = −0.015; *p* = 0.774	−0.09 [−0.77, 0.60] β = −0.012; *p* = 0.804
Retired (ref: working)	−3.53 [−8.53, 1.47] β = −0.084; *p* = 0.166	0.35 [−4.51, 5.22] β = 0.008; *p* = 0.887	−0.90 [−5.99, 4.18] β = −0.021; *p* = 0.727
Not working (ref: working)	−2.14 [−8.45, 4.18] β = −0.046; *p* = 0.506	−2.07 [−7.87, 3.74] β = −0.045; *p* = 0.485	−2.02 [−8.22, 4.19] β = −0.043; *p* = 0.523
Rural residence (ref: urban)	1.03 [−2.77, 4.83] β = 0.024; *p* = 0.595	−1.11 [−4.77, 2.54] β = −0.026; *p* = 0.550	0.46 [−3.42, 4.34] β = 0.011; *p* = 0.817
Unmarried (ref: married)	−3.80 [−12.60, 5.00] β = −0.045; *p* = 0.396	0.86 [−6.47, 8.19] β = 0.010; *p* = 0.817	1.87 [−7.97, 11.72] β = 0.022; *p* = 0.709
Current smoking (ref: no)	−2.76 [−6.87, 1.34] β = −0.066; *p* = 0.186	−6.69 [−10.78, −2.60] β = −0.160; *p* = 0.001	−3.74 [−7.99, 0.51] β = −0.088; *p* = 0.085
Not physically active (ref: active)	−2.35 [−6.88, 2.17] β = −0.057; *p* = 0.307	−0.19 [−4.56, 4.17] β = −0.005; *p* = 0.930	0.83 [−3.57, 5.23] β = 0.020; *p* = 0.712
Sleeping difficulty (ref: no)	−1.79 [−6.09, 2.52] β = −0.043; *p* = 0.415	−3.28 [−7.59, 1.03] β = −0.079; *p* = 0.135	−4.13 [−8.52, 0.27] β = −0.098; *p* = 0.066
HF duration < 1 year (ref: diagnosed at visit)	1.69 [−4.81, 8.18] β = 0.034; *p* = 0.610	−3.61 [−9.58, 2.36] β = −0.074; *p* = 0.236	−2.13 [−8.30, 4.03] β = −0.043; *p* = 0.496
HF duration ≥ 1 year (ref: diagnosed at visit)	0.86 [−4.99, 6.72] β = 0.020; *p* = 0.772	−1.48 [−7.00, 4.05] β = −0.035; *p* = 0.600	−2.77 [−8.22, 2.68] β = −0.064; *p* = 0.319
No prior HF admission (ref: yes)	−2.68 [−6.85, 1.50] β = −0.064; *p* = 0.209	−4.32 [−8.21, −0.42] β = −0.104; *p* = 0.030	−3.35 [−7.43, 0.73] β = −0.080; *p* = 0.107
HF knowledge, per 10 percentage points	2.02 [0.50, 3.54] β = 0.128; *p* = 0.009	2.26 [0.78, 3.73] β = 0.143; *p* = 0.003	3.81 [2.32, 5.30] β = 0.237; *p* < 0.001
HADS anxiety, 0–21	0.55 [−0.10, 1.19] β = 0.114; *p* = 0.095	1.37 [0.74, 2.00] β = 0.288; *p* < 0.001	0.92 [0.33, 1.51] β = 0.189; *p* = 0.002
HADS depression, 0–21	−0.94 [−1.57, −0.31] β = −0.188; *p* = 0.004	−1.47 [−2.10, −0.83] β = −0.296; *p* < 0.001	−1.09 [−1.70, −0.48] β = −0.216; *p* < 0.001
Model fit	N = 516; R^2^ = 0.119; adjusted R^2^ = 0.076 HC3 F(24, 491) = 2.96; *p* < 0.001	N = 516; R^2^ = 0.148; adjusted R^2^ = 0.107 HC3 F(24, 491) = 3.42; *p* < 0.001	N = 516; R^2^ = 0.130; adjusted R^2^ = 0.088 HC3 F(24, 491) = 3.24; *p* < 0.001
**Panel B. Separate Self-Care Confidence model**			
**Predictor**		**Confidence** **B [95% CI]; β; *p***	
Age, years		0.20 [−0.06, 0.45] β = 0.074; *p* = 0.138	
Female (ref: male)		1.71 [−4.59, 8.01] β = 0.027; *p* = 0.594	
BMI, kg/m^2^		0.39 [−0.04, 0.82] β = 0.092; *p* = 0.076	
NYHA class II (ref: I)		−0.53 [−6.01, 4.95] β = −0.011; *p* = 0.849	
NYHA class III (ref: I)		1.88 [−4.47, 8.24] β = 0.035; *p* = 0.561	
HFmrEF, LVEF 41–49% (ref: HFpEF)		1.58 [−6.16, 9.33] β = 0.030; *p* = 0.688	
HFrEF, LVEF ≤ 40% (ref: HFpEF)		−1.20 [−9.07, 6.67] β = −0.024; *p* = 0.764	
No education (ref: higher)		−3.47 [−15.42, 8.47] β = −0.031; *p* = 0.568	
Elementary education (ref: higher)		1.15 [−6.61, 8.92] β = 0.015; *p* = 0.770	
Secondary education (ref: higher)		1.33 [−3.75, 6.42] β = 0.026; *p* = 0.607	
Monthly income, per 100 JD		−0.61 [−1.33, 0.10] β = −0.074; *p* = 0.094	
Retired (ref: working)		2.34 [−3.48, 8.16] β = 0.047; *p* = 0.429	
Not working (ref: working)		0.53 [−6.03, 7.10] β = 0.010; *p* = 0.873	
Rural residence (ref: urban)		−2.97 [−7.26, 1.32] β = −0.058; *p* = 0.174	
Unmarried (ref: married)		−0.20 [−9.98, 9.58] β = −0.002; *p* = 0.967	
Current smoking (ref: no)		−3.11 [−7.70, 1.48] β = −0.062; *p* = 0.183	
Not physically active (ref: active)		3.05 [−1.86, 7.96] β = 0.062; *p* = 0.223	
Sleeping difficulty (ref: no)		−4.10 [−9.09, 0.89] β = −0.082; *p* = 0.107	
HF duration < 1 year (ref: diagnosed at visit)		−2.54 [−9.36, 4.27] β = −0.044; *p* = 0.464	
HF duration ≥ 1 year (ref: diagnosed at visit)		−3.94 [−9.89, 2.01] β = −0.078; *p* = 0.194	
No prior HF admission (ref: yes)		2.13 [−2.62, 6.88] β = 0.043; *p* = 0.379	
HF knowledge, per 10 percentage points		3.58 [1.88, 5.28] β = 0.190; *p* < 0.001	
HADS anxiety, 0–21		0.75 [0.05, 1.44] β = 0.131; *p* = 0.036	
HADS depression, 0–21		−2.22 [−2.92, −1.52] β = −0.373; *p* < 0.001	
Model fit		N = 516; R^2^ = 0.168; adjusted R^2^ = 0.127 HC3 F(24, 491) = 4.93; *p* < 0.001	

Note. B = unstandardized coefficient; β = standardized coefficient; CI = HC3 95% confidence interval; HC3 = heteroscedasticity-consistent robust standard error type 3. Income coefficients are expressed per 100 JD and HF-knowledge coefficients per 10 percentage points. Reference categories are stated in the predictor labels. All models used N = 516 and residual df = 491.

**Table 4:** Association rules for SCHFI behavioral-scale scores < 70. The separate Self-Care Confidence outcome was evaluated under the same criteria.

Antecedent Profile	SCHFI Scale	Profile n	Score < 70 n	Rule Confidence, %	Lift	Prevalence Difference, pp	Mean Score Difference
Male; no prior HF admission; HADS depression 11–21	Maintenance	42	40	95.2	1.199	17.2	−5.96
Education ≤ secondary; current smoking; no prior HF admission	Maintenance	57	53	93.0	1.170	15.2	−11.00
Male; NYHA class III; HF knowledge 50–<60%	Maintenance	41	38	92.7	1.166	14.4	−7.56
Male; sleeping difficulty; HF knowledge 50–<60%	Management	51	50	98.0	1.246	21.5	−7.85
Current smoking; HF knowledge 50–<60%	Management	72	68	94.4	1.200	18.3	−7.61
BMI < 30 kg/m^2^; NYHA class III; sleeping difficulty	Management	48	45	93.8	1.192	16.6	−6.97
NYHA class III; education ≤ secondary; current smoking	Management	44	41	93.2	1.184	15.9	−8.09
LVEF > 40%; current smoking; no prior HF admission	Symptom perception	53	52	98.1	1.143	13.7	−9.63
Age ≥ 65 years; HF knowledge < 50%	Symptom perception	51	50	98.0	1.142	13.5	−5.43
Male; LVEF > 40%; HADS depression 8–10	Symptom perception	50	49	98.0	1.141	13.5	−10.05
Self-Care Confidence: no profile met the established rule-retention criteria.							

Note. N = 516. Confidence is the proportion of participants satisfying the antecedent profile who had the indicated SCHFI score. Lift compares confidence with the corresponding outcome prevalence in the full sample. Prevalence difference compares participants inside versus outside the antecedent profile. Mean score difference is the profile-group mean minus the mean among all other participants. All retained rules had support ≥ 5%, at least 40 participants in the profile, at least 26 participants satisfying both profile and outcome, a 5th-percentile lift > 1.10 across 500 random 20% case-deletion repetitions, and a continuous-score difference of at least 5 points. In this table, rule confidence is an association-rule metric and is distinct from the SCHFI Self-Care Confidence score.

## Data Availability

The study data are available from the corresponding authors upon reasonable request.

## Supplementary Materials

The following supporting information can be downloaded at: https://www.mdpi.com/article/10.3390/healthcare14172772/s1, Figure S1: OLS diagnostic plots for Maintenance; Figure S2: OLS diagnostic plots for Symptom Perception; Figure S3: OLS diagnostic plots for Management; Figure S4: OLS diagnostic plots for Self-Care Confidence; Table S1: Model samples, fit, and HC3 overall omnibus tests; Table S2: Residual, heteroscedasticity, specification, and collinearity checks; Table S3: Influence screening; Table S4: Joint HC3 tests of standardized quadratic terms for six continuous predictors; Table S5: Floor and ceiling frequencies; Table S6: Group-specific SCHFI distributions and Kruskal–Wallis tests; Table S7: Dunn comparisons following the Maintenance LVEF omnibus test; Table S8: LVEF phenotype distribution by sex; Table S9: Model fit and HC3 overall omnibus tests for the content-overlap sensitivity models; Table S10: HC3 regression estimates from the content-overlap sensitivity models.
