# Supplementary material for: Psychosocial, Clinical, and Knowledge-Based Factors Associated with Self-Care in Heart Failure: A Cross-Sectional Study
Source: Healthcare (Basel). 2026 Sep 1;14(17):2772. doi: 10.3390/healthcare14172772 (PMC13565915; doi:10.3390/healthcare14172772)
Supplement: Supplementary file 1 [file healthcare-14-02772-s001.zip › healthcare-4431860-supplementary.pdf]

Supplementary File S1. Statistical Diagnostics and Group-Specific Results

This file reports the diagnostic and descriptive outputs for the three ordinary least-squares models of SCHFI Maintenance, Symptom Perception, and Management. All three models used N=516, 24 predictor degrees of freedom, residual df=491, and complete model frames. It also reports the separate Self-Care Confidence model, which used N=516, 24 predictor degrees of freedom, and residual df=491.

Table S1. Model samples, fit, and HC3 overall omnibus tests.

Outcome	N	Predictor df	Residual df	R^2	Adjusted R^2	HC3 F	p
Maintenance	516	24	491	0.119	0.076	2.96	<.001
Symptom Perception	516	24	491	0.148	0.107	3.42	<.001
Management	516	24	491	0.130	0.088	3.24	<.001
Self-Care Confidence	516	24	491	0.168	0.127	4.93	<.001

Table S2. Residual, heteroscedasticity, specification, and collinearity checks.

Outcome	BP χ^2	BP p	Shapiro W	Shapiro p	Skewness	Excess kurtosis	RESET p	Max adj. GVIF
Maintenance	39.73	.023	0.993	.016	0.256	-0.269	.045	1.448
Symptom Perception	15.55	.903	0.988	<.001	0.243	-0.518	.576	1.448
Management	38.49	.031	0.995	.110	0.022	-0.467	.457	1.448
Self-Care Confidence	34.81	.071	0.987	<.001	−0.292	−0.480	.596	1.448

Table S3. Influence screening.

Outcome	|rstudent|>3, n	Cook D>1, n	Cook D>4/N, n	Maximum Cook D	Leverage>2p/N, n	Maximum leverage
Maintenance	1	0	27	0.034	9	0.206
Symptom Perception	0	0	21	0.029	9	0.206
Management	0	0	27	0.039	9	0.206
Self-Care Confidence	1	0	25	0.038	9	0.206

Note. The leverage threshold was 2p/N with p=25 coefficients including the intercept (0.0969). Cook's D>4/N was used as a screening threshold and not as an automatic exclusion rule. No Cook's distance exceeded 1.

Table S4. Joint HC3 tests of standardized quadratic terms for six continuous predictors.

Outcome	df1	df2	F	p
Maintenance	6	485	1.40	.215
Symptom Perception	6	485	0.14	.991
Management	6	485	1.36	.228
Self-Care Confidence	6	485	1.15	.331

The isolated Maintenance BMI quadratic term in the full diagnostic model was sensitive to the single maximum-BMI observation. After excluding that observation only from the diagnostic refit, its Holm-adjusted p-value was 1.000 and the joint six-term p-value was 0.931; the primary model retained all 516 participants.

Table S5. Floor and ceiling frequencies.

Outcome	N	Observed range	Floor, n (%)	Ceiling, n (%)
Maintenance	516	0.00–100.00	3 (0.58%)	2 (0.39%)
Symptom Perception	516	0.00–95.65	3 (0.58%)	0 (0.00%)
Management	516	0.00–100.00	2 (0.39%)	2 (0.39%)
Self-Care Confidence	516	0.00–100.00	7 (1.36%)	36 (6.98%)

Table S6. Group-specific SCHFI distributions and Kruskal–Wallis tests.

Grouping	Outcome	Group	n	Median (IQR)	H	df	p
NYHA	Maintenance	I	104	51.25 (36.25–66.25)	3.24	2	.198
NYHA	Maintenance	II	258	50.00 (35.00–65.00)			
NYHA	Maintenance	III	154	45.00 (30.00–62.50)			

Grouping	Outcome	Group	n	Median (IQR)	H	df	p
NYHA	Symptom Perception	I	104	39.13 (24.39–56.52)	4.47	2	.107
NYHA	Symptom Perception	II	258	43.48 (32.61–60.87)			
NYHA	Symptom Perception	III	154	46.00 (34.78–61.11)			
NYHA	Management	I	104	51.52 (39.39–69.70)	0.74	2	.691
NYHA	Management	II	258	54.55 (36.36–69.70)			
NYHA	Management	III	154	51.52 (39.39–69.70)			
NYHA	Confidence	I	104	70.00 (51.25–87.50)	3.63	2	.163
NYHA	Confidence	II	258	67.50 (47.50–87.50)			
NYHA	Confidence	III	154	62.50 (50.00–82.50)			
LVEF	Maintenance	HFpEF (≥50%)	47	50.00 (35.00–62.50)	6.12	2	.047
LVEF	Maintenance	HFmrEF (41–49%)	168	50.00 (37.50–68.75)			
LVEF	Maintenance	HFrEF (≤40%)	301	47.50 (32.50–62.50)			
LVEF	Symptom Perception	HFpEF (≥50%)	47	39.13 (23.91–52.17)	2.11	2	.347
LVEF	Symptom Perception	HFmrEF (41–49%)	168	42.39 (32.61–60.87)			
LVEF	Symptom Perception	HFrEF (≤40%)	301	44.44 (30.43–61.11)			
LVEF	Management	HFpEF (≥50%)	47	57.58 (39.39–75.76)	1.04	2	.596
LVEF	Management	HFmrEF (41–49%)	168	54.55 (42.42–69.70)			
LVEF	Management	HFrEF (≤40%)	301	51.52 (36.36–69.70)			
LVEF	Confidence	HFpEF (≥50%)	47	70.00 (50.00–87.50)	1.74	2	.418
LVEF	Confidence	HFmrEF (41–49%)	168	68.75 (50.00–85.00)			
LVEF	Confidence	HFrEF (≤40%)	301	65.00 (45.00–87.50)			

Table S7. Dunn comparisons following the Maintenance LVEF omnibus test.

Group 1	Group 2	z	Unadjusted p	Holm-adjusted p
>=50%	41-49%	-0.38	.705	.705
>=50%	<=40%	1.08	.280	.560
41-49%	<=40%	2.41	.016	.048

Table S8. LVEF phenotype distribution by sex.

Sex	LVEF phenotype	n	% within sex
Male	HFpEF	37	8.9%
Female	HFpEF	10	10.2%
Male	HFmrEF	136	32.5%
Female	HFmrEF	32	32.7%
Male	HFrEF	245	58.6%
Female	HFrEF	56	57.1%

Diagnostic interpretation. Breusch–Pagan tests indicated heteroscedasticity for Maintenance and Management, and Shapiro–Wilk tests indicated residual-distribution departures for Maintenance and Symptom Perception. The Maintenance RESET p-value was 0.045. These findings were considered together with the diagnostic plots, negligible floor/ceiling frequencies, nonsignificant joint quadratic tests, low adjusted GVIF values, and absence of a dominant influential observation. HC3 inference was retained for all primary coefficient and omnibus tests. For Self-Care Confidence, the diagnostic results were: Breusch–Pagan p=0.071, Shapiro–Wilk p<0.001, RESET p=0.596, joint quadratic-term p=0.331, and maximum adjusted GVIF=1.448.

Figure S1. OLS diagnostic plots for Maintenance.

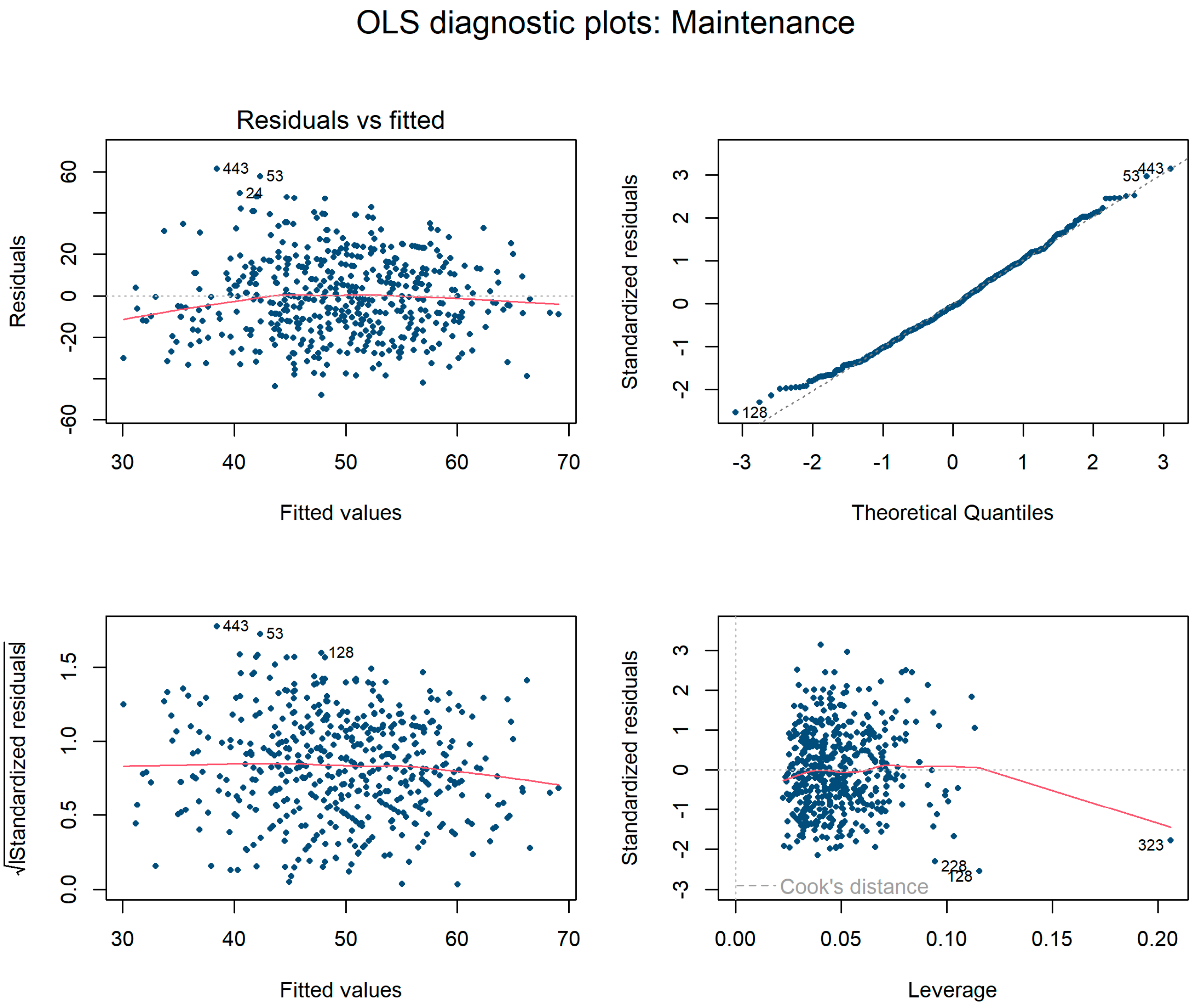

Figure S2. OLS diagnostic plots for Symptom Perception.

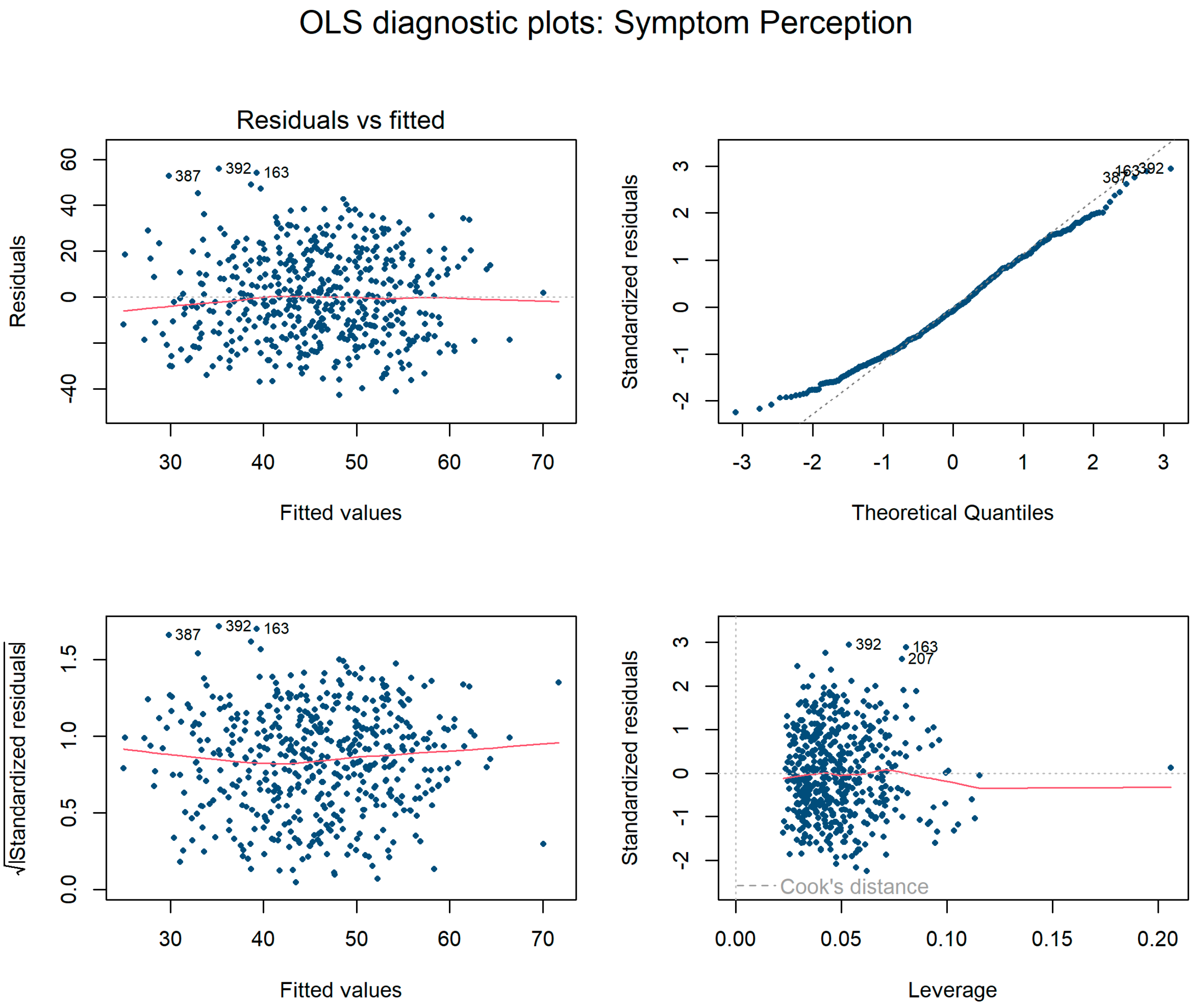

Figure S3. OLS diagnostic plots for Management.

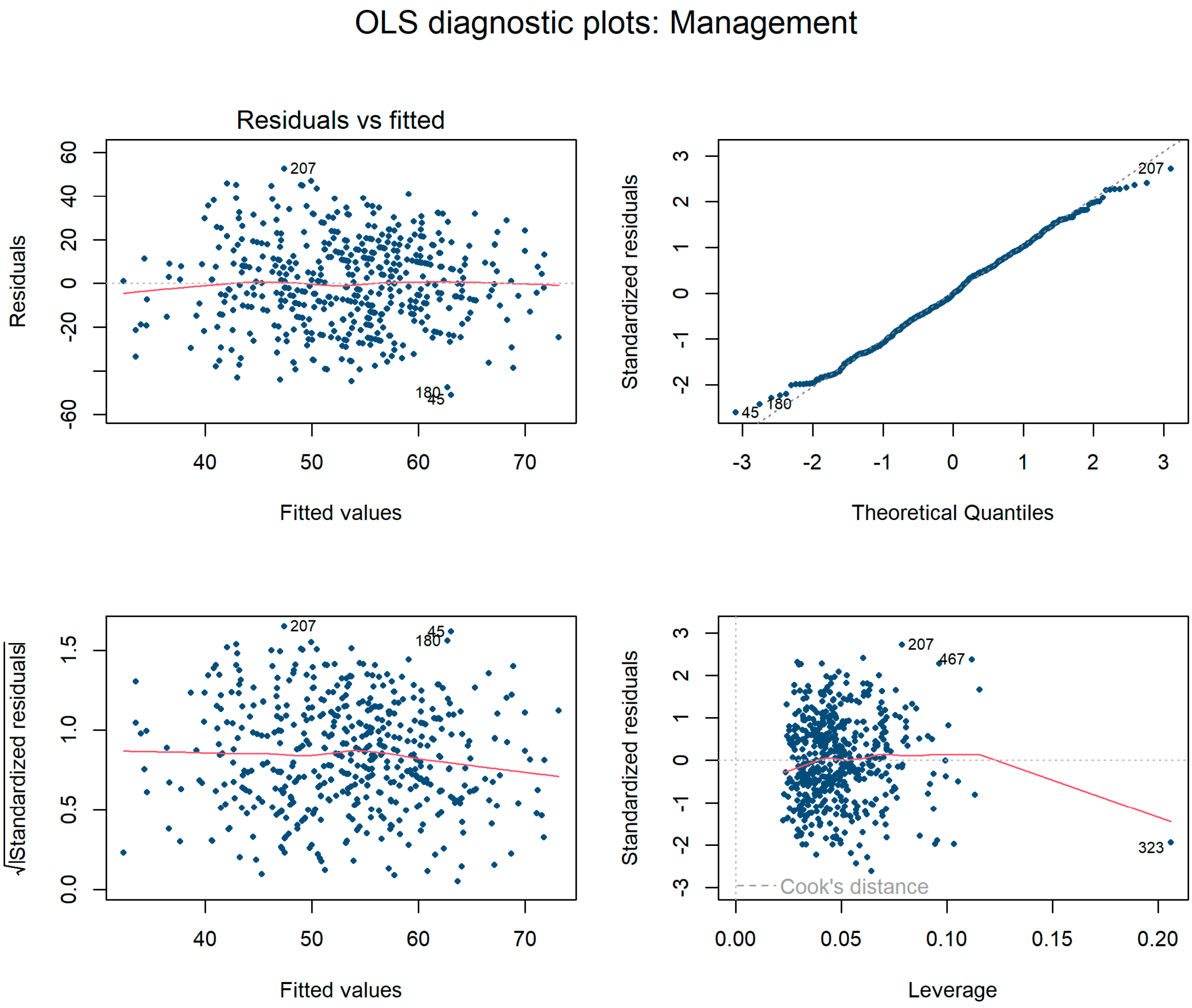

Figure S4. OLS diagnostic plots for Self-Care Confidence.

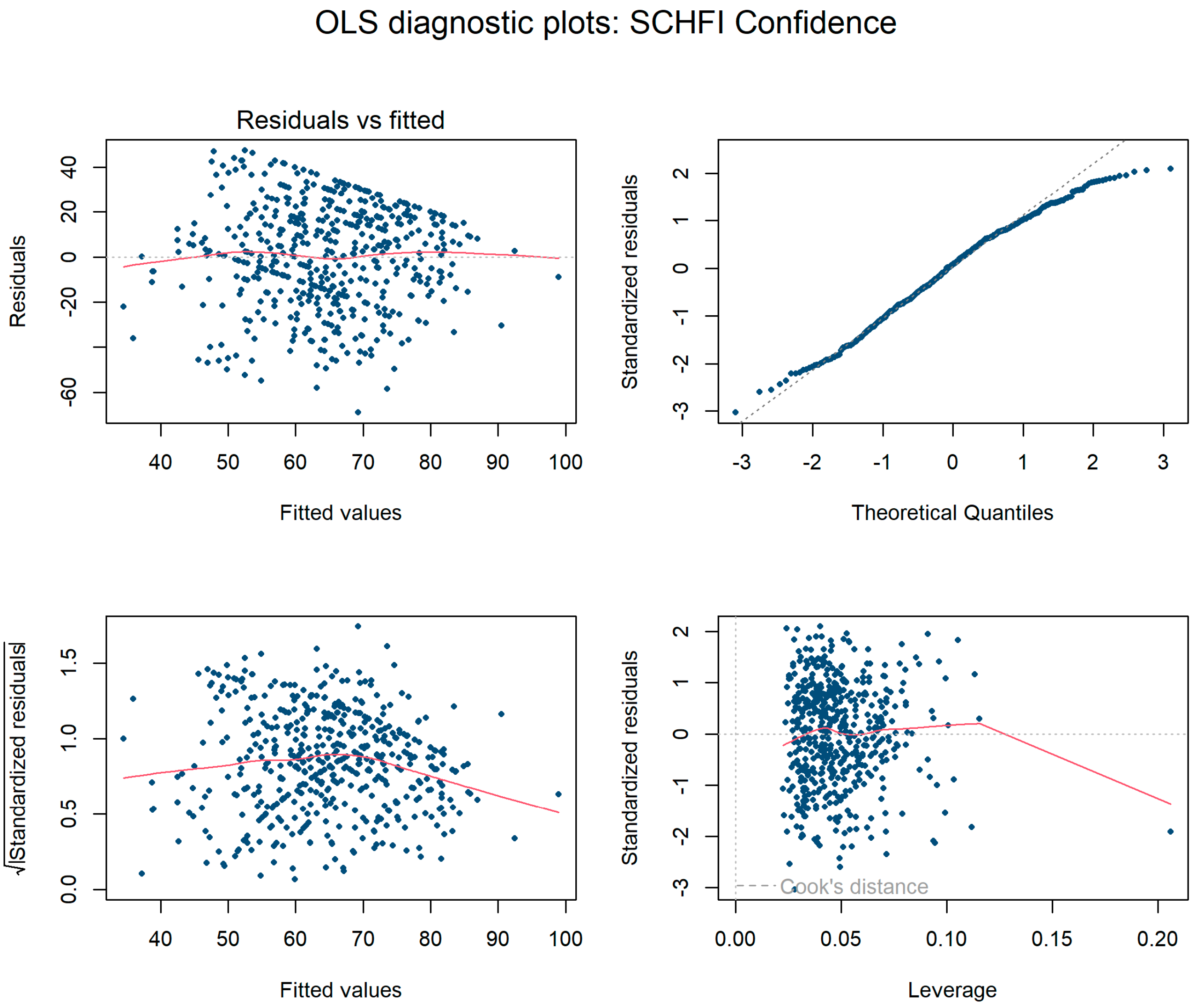

Sensitivity analysis for conceptual overlap

Sensitivity models were used to assess whether associations involving the remaining covariates depended on inclusion of measures with direct content overlap. HF knowledge was omitted from all three models, and physical activity was additionally omitted from the Maintenance model. The same 516 participants and HC3 procedures were retained. The Self-Care Confidence sensitivity model omitted HF knowledge and retained the remaining covariates.

Table S9. Model fit and HC3 overall omnibus tests for the content-overlap sensitivity models.

Outcome	N	Predictor df	Residual df	R^2	Adjusted R^2	HC3 F	p
Maintenance	516	22	493	0.101	0.061	2.70	<.001
Symptom Perception	516	23	492	0.130	0.089	3.14	<.001
Management	516	23	492	0.080	0.037	1.77	.015
Self-Care Confidence	516	23	492	0.136	0.096	4.06	<.001

Note. The Maintenance model excluded HF knowledge and physical activity; the Symptom Perception and Management models excluded HF knowledge. All models used N=516 and HC3 robust inference. The Self-Care Confidence model excluded HF knowledge.

Table S10. HC3 regression estimates from the content-overlap sensitivity models.

Predictor	Maintenance B [95% CI]; β; p	Symptom Perception B [95% CI]; β; p	Management B [95% CI]; β; p
Age, years	0.10 [−0.13, 0.33] β=.046; p=.385	0.04 [−0.18, 0.25] β=.016; p=.741	0.15 [−0.09, 0.39] β=.069; p=.209
Female (ref: male)	4.45 [−1.13, 10.02] β=.084; p=.118	5.09 [−0.67, 10.84] β=.097; p=.083	4.07 [−1.78, 9.91] β=.076; p=.172
BMI, kg/m²	0.01 [−0.38, 0.41] β=.004; p=.942	0.24 [−0.05, 0.53] β=.068; p=.100	0.27 [−0.16, 0.70] β=.076; p=.213
NYHA class II (ref: I)	−0.31 [−5.39, 4.76] β=−.008; p=.903	4.03 [−0.80, 8.87] β=.098; p=.102	3.07 [−1.91, 8.05] β=.073; p=.226
NYHA class III (ref: I)	−0.95 [−6.85, 4.95] β=−.021; p=.751	5.92 [0.10, 11.73] β=.131; p=.046	4.77 [−1.06, 10.59] β=.104; p=.109
HFmrEF, LVEF 41–49% (ref: HFpEF)	2.66 [−4.27, 9.59] β=.060; p=.451	5.97 [−1.83, 13.77] β=.135; p=.133	2.25 [−4.96, 9.45] β=.050; p=.540
HFrEF, LVEF ≤40% (ref: HFpEF)	−0.56 [−7.28, 6.15] β=−.013; p=.869	6.17 [−1.51, 13.86] β=.147; p=.115	0.33 [−6.74, 7.41] β=.008; p=.926
No education (ref: higher)	−3.43 [−14.81, 7.95] β=−.036; p=.554	−4.52 [−13.50, 4.46] β=−.048; p=.323	−1.23 [−12.74, 10.27] β=−.013; p=.833
Elementary education (ref: higher)	−11.10 [−17.40, −4.79] β=−.170; p<.001	−1.71 [−8.13, 4.71] β=−.026; p=.601	−5.35 [−12.35, 1.66] β=−.081; p=.134
Secondary education (ref: higher)	−6.70 [−11.24, −2.16] β=−.158; p=.004	−0.75 [−5.30, 3.81] β=−.018; p=.747	−1.81 [−6.59, 2.98] β=−.042; p=.458
Monthly income, per 100 JD	−0.19 [−0.87, 0.48] β=−.028; p=.569	0.05 [−0.65, 0.75] β=.007; p=.891	0.17 [−0.54, 0.87] β=.024; p=.646
Retired (ref: working)	−4.04 [−9.07, 0.98] β=−.096; p=.114	0.01 [−4.84, 4.86] β=.000; p=.997	−1.48 [−6.61, 3.64] β=−.035; p=.570
Not working (ref: working)	−2.39 [−8.69, 3.91] β=−.052; p=.457	−2.02 [−7.88, 3.83] β=−.044; p=.498	−1.95 [−8.26, 4.37] β=−.042; p=.545

Predictor	Maintenance B [95% CI]; β; p	Symptom Perception B [95% CI]; β; p	Management B [95% CI]; β; p
Rural residence (ref: urban)	1.17 [−2.64, 4.99] β=.027; p=.547	−1.04 [−4.70, 2.61] β=−.025; p=.576	0.57 [−3.36, 4.50] β=.013; p=.774
Unmarried (ref: married)	−3.75 [−12.53, 5.03] β=−.045; p=.402	1.30 [−6.17, 8.77] β=.016; p=.733	2.61 [−7.56, 12.79] β=.031; p=.614
Current smoking (ref: no)	−2.92 [−7.02, 1.17] β=−.070; p=.162	−6.91 [−11.03, −2.79] β=−.165; p=.001	−4.11 [−8.41, 0.20] β=−.096; p=.061
Not physically active (ref: active)	Excluded	−0.82 [−5.14, 3.51] β=−.020; p=.711	−0.22 [−4.75, 4.30] β=−.005; p=.923
Sleeping difficulty (ref: no)	−2.14 [−6.34, 2.06] β=−.051; p=.318	−3.17 [−7.51, 1.16] β=−.076; p=.151	−3.94 [−8.40, 0.53] β=−.093; p=.084
HF duration <1 year (ref: diagnosed at visit)	1.36 [−5.19, 7.90] β=.028; p=.684	−4.18 [−10.28, 1.92] β=−.086; p=.179	−3.10 [−9.50, 3.30] β=−.062; p=.342
HF duration ≥1 year (ref: diagnosed at visit)	0.93 [−4.97, 6.82] β=.022; p=.758	−1.36 [−7.00, 4.27] β=−.032; p=.635	−2.58 [−8.21, 3.05] β=−.060; p=.368
No prior HF admission (ref: yes)	−2.01 [−6.12, 2.10] β=−.048; p=.338	−4.19 [−8.16, −0.21] β=−.101; p=.039	−3.13 [−7.39, 1.12] β=−.074; p=.148
HF knowledge, per 10 percentage points	Excluded	Excluded	Excluded
HADS anxiety, 0–21	0.51 [−0.13, 1.15] β=.107; p=.115	1.34 [0.72, 1.97] β=.282; p<.001	0.87 [0.27, 1.46] β=.179; p=.004
HADS depression, 0–21	−1.05 [−1.69, −0.40] β=−.210; p=.001	−1.50 [−2.14, −0.86] β=−.302; p<.001	−1.14 [−1.76, −0.52] β=−.226; p<.001
**Model fit**	**N=516; R^2=0.101; adjusted R^2=0.061; HC3 F(22,493)=2.70; p<.001**	**N=516; R^2=0.130; adjusted R^2=0.089; HC3 F(23,492)=3.14; p<.001**	**N=516; R^2=0.080; adjusted R^2=0.037; HC3 F(23,492)=1.77; p=.015**

Panel B. Separate Self-Care Confidence sensitivity model

Predictor	Confidence B [95% CI]; β; p
Age, years	0.19 [−0.07, 0.46] β=.074; p=.151
Female (ref: male)	2.10 [−4.44, 8.65] β=.033; p=.528
BMI, kg/m²	0.37 [−0.09, 0.83] β=.087; p=.116
NYHA class II (ref: I)	0.37 [−5.14, 5.88] β=.007; p=.895
NYHA class III (ref: I)	2.08 [−4.36, 8.52] β=.038; p=.526
HFmrEF, LVEF 41–49% (ref: HFpEF)	2.62 [−5.28, 10.51] β=.050; p=.515
HFrEF, LVEF ≤40% (ref: HFpEF)	0.50 [−7.44, 8.44] β=.010; p=.901
No education (ref: higher)	−6.12 [−18.40, 6.16] β=−.054; p=.328
Elementary education (ref: higher)	−0.43 [−8.22, 7.37] β=−.005; p=.915
Secondary education (ref: higher)	0.51 [−4.70, 5.71] β=.010; p=.849

Panel B. Separate Self-Care Confidence sensitivity model	
**Predictor**	**Confidence B [95% CI]; β; p**
Monthly income, per 100 JD	−0.37 [−1.09, 0.34] β=−.045; p=.302
Retired (ref: working)	1.80 [−4.03, 7.63] β=.036; p=.545
Not working (ref: working)	0.60 [−6.11, 7.31] β=.011; p=.860
Rural residence (ref: urban)	−2.86 [−7.20, 1.48] β=−.056; p=.196
Unmarried (ref: married)	0.49 [−9.70, 10.69] β=.005; p=.924
Current smoking (ref: no)	−3.46 [−8.09, 1.17] β=−.069; p=.143
Not physically active (ref: active)	2.06 [−2.95, 7.07] β=.042; p=.420
Sleeping difficulty (ref: no)	−3.92 [−8.98, 1.14] β=−.079; p=.128
HF duration <1 year (ref: diagnosed at visit)	−3.45 [−10.57, 3.68] β=−.059; p=.342
HF duration ≥1 year (ref: diagnosed at visit)	−3.76 [−9.94, 2.42] β=−.075; p=.232
No prior HF admission (ref: yes)	2.33 [−2.55, 7.21] β=.047; p=.348
HF knowledge, per 10 percentage points	Excluded
HADS anxiety, 0–21	0.70 [−0.01, 1.41] β=.123; p=.053
HADS depression, 0–21	−2.27 [−2.99, −1.55] β=−.381; p<.001
**Model fit**	**N=516; R^2=0.136; adjusted R^2=0.096 HC3 F(23,492)=4.06; p<.001**

Note. B=unstandardized coefficient; β=standardized coefficient; CI=HC3 95% confidence interval; HC3=heteroscedasticity-consistent robust standard error type 3. Excluded indicates a predictor omitted by design because of direct content overlap. Income coefficients are expressed per 100 JD. Reference categories are stated in the predictor labels. No retained predictor changed statistical significance relative to the corresponding primary model. Panel B reports the separate Self-Care Confidence sensitivity model. The statement concerning unchanged statistical significance applies to the three behavioral models. In the Confidence sensitivity model excluding HF knowledge, HADS anxiety, 0–21 (B=0.70, 95% CI [−0.01, 1.41], p=.053) no longer met the p<0.05 threshold. No other retained Confidence predictor crossed that threshold.
